# An embryo lethal transgenic line manifests global expression changes and elevated protein/oil ratios in heterozygous soybean plants

**DOI:** 10.1371/journal.pone.0233721

**Published:** 2020-06-09

**Authors:** Sarah I. Jones, Matt R. Hunt, Lila O. Vodkin

**Affiliations:** Department of Crop Sciences, University of Illinois, Urbana, Illinois, United States of America; Agriculture and Agri-Food Canada, CANADA

## Abstract

Understanding the molecular processes of seed development is important especially in agronomic crops that produce large amounts of nutrient reserves. Because soybean is a vital source of vegetable protein worldwide, producers are concerned about increasing the total amount of protein in the seed without substantially lowering the amount of oil, another economically important product. Here we describe a transgenic soybean line with increased protein and protein/oil ratio, containing an average of 42.2% protein vs. 38.5% in controls and with a protein/oil ratio of 2.02 vs. 1.76 in controls over several generations of greenhouse growth. Other phenotypic data show that the seeds are heavier, although there are overall lower yields per plant. We postulate these effects result from insertion site mutagenesis by the transgenic construct. As this line never achieves homozygosity and appears to be embryo lethal when homozygous, one functional copy of the gene is most likely essential for normal seed development. Global transcript analyses using RNA-Seq for 88,000 gene models over two stages of cotyledon development revealed that more genes are over-expressed in the transgenic line including ribosomal protein related genes and those in the membrane protein and transporters families. Localization of the insertion site should reveal the genes and developmental program that has been perturbed by the transgenic construct, resulting in this economically interesting increase in protein and the protein/oil ratio.

## Introduction

Soybean is one of the most important sources of vegetable protein and oil in the world. In the US alone, over 88 million acres of soybeans were harvested in 2018 [1], with production worth almost $40 billion [2]. About 70% of this soybean production goes towards animal feed [3] due to its broad availability and high protein content. In addition its amino acid content complements that of a grain such as corn, providing nearly complete nutrition for livestock [4]. Thus, soy is a major source of animal feed. Soybean protein can also be consumed directly by humans (as in tofu or protein powders) and may have health benefits such as lowering cholesterol [5]. Soy protein is frequently found as an additive in a wide variety of other human foods such as bakery products and processed meats due to its favorable industrial qualities, which include the ability to increase protein without adding fat and to retain moisture and texture; additionally, soy protein is generally easy to digest and contains all essential amino acids for humans [6]. Soy protein is also being investigated as an industrial material for biomedical uses, for example drug delivery and tissue engineering [7]. In nature, these soy proteins are mostly storage proteins found in the mature, dry seed and later used by the germinating seedlings as a food reserve until they can support themselves with photosynthesis. These proteins are mainly glycinin and beta-conglycinin, part of the globulin family. The glycinin and beta-conglycinin soybean proteins have been extensively studied [e.g., 8, 9, 10], as have efforts to alter the amount or composition of the proteins [11]. The process of seed development is especially important to the production of protein and oil, as during this time, changes in gene expression in the immature seeds affect the amount and composition of the final products. Soybean seed development has frequently been the target of large-scale gene expression studies [e.g., 12, 13, 14] but it is a complex process and many unknowns remain regarding how the flux of protein and oil production is controlled over time.

Here, we analyzed a transgenic soybean line with elevated protein and a higher protein/oil ratio than control plants. The transgene construct was designed as a siRNA down-regulation vector, but it did not produce small RNAs or the expected decrease in expression of the target gene. However, the higher protein production, with a disproportionately small drop in oil production, warranted further investigation. It is speculated that the transgenic insertion disrupted the function of a developmentally important gene, leading to the increased protein content and to other unusual growth characteristics. The agronomic properties of these greenhouse-grown plants were studied over multiple generations, using phenotypic data including near-infrared (NIR) imaging to determine protein and oil content in a non-destructive manner. Additionally, high-throughput RNA-Seq data for 88,000 gene models were obtained from multiple plants across two stages of immature seed development. The lines displayed abnormal segregation of the transgene, never becoming homozygous, suggesting that having two copies of the disrupted gene might be lethal. Characterizing this line may prove useful for further elucidating how protein and oil production are regulated during soybean seed development and to provide opportunities to alter the amount or composition of the protein for various end products.

## Results

A seed-targeted transgenic construct based on RNA interference (RNAi) was produced (S1 Fig) and introduced into soybean tissue in an attempt to down-regulate the pyruvate dehydrogenase kinase (PDHK) gene. This would theoretically increase the activity of the mitochondrial pyruvate dehydrogenase complex (PDC) during the day, shuttling more carbon into the tricarboxylic acid (TCA/Krebs/citric acid) cycle and thus producing more ATP and amino acid precursors and increasing the final seed weight. Soybean embryogenic culture pieces were transformed with this construct and some regenerated plants that were positive for the hygromycin gene of the construct via PCR and Southern blots were selected [15]. Additionally, homozygous lines could not be established as no future generations bred true, with some offspring positive for the transgenic construct and some negative. The subline selected for future studies was based on the finding of a higher average weight per 100 seeds and a higher protein/oil ratio and represented only a single event.

**Fig 1:**
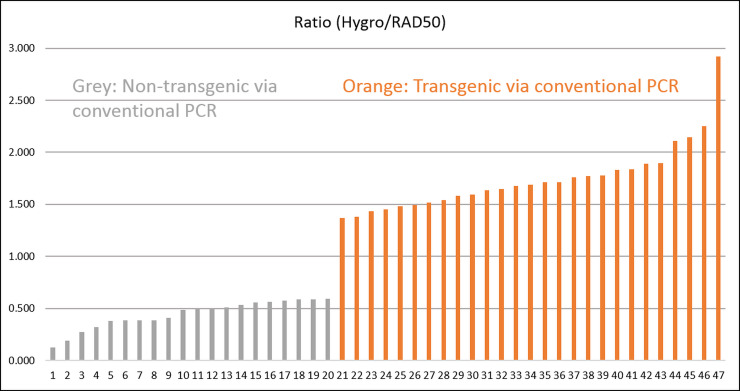
Ratio of hygromycin amplification/RAD50 control gene amplification in digital pcr, ordered low to high. Digital PCR data show a clear distinction between the two groups that agrees with conventional PCR data. Samples in grey were found to be non-transgenic segregant plants via digital PCR (negative); samples in orange were found to be transgenic (positive). See S1 Table for list of plants used and ratios, and S2 Fig for example amplification graphs.

Here, we examined molecular and agronomic properties of three subsequent generations of plants from this single line by digital PCR (dPCR) (Fig 1, S2 Fig, S1 Table and S2 Table) in addition to conventional PCR (S3 Table and S4 Table, S3 Fig). A few plants were also subjected to high-throughput RNA sequencing and those predicted to be transgenic contained transcripts matching hygromycin which was part of the transgenic construct (S2 Table and S5 Table).

PDHK gene expression in the initial generations of transgenic plants was not significantly different from that observed in the Jack control plants, as determined by Northern blots and qRT-PCR [15]. In order to determine whether short interfering RNAs were produced, we performed small RNA sequencing on some of the same plant total RNA samples that proved positive for hygromycin by RNA-Seq analyses. Analysis of the results revealed a peak of thousands of small RNAs in the range of 18–25 nt but few siRNAs that aligned to the PDHK gene (S6 Table). Out of millions of small RNA high-throughput sequencing reads, only a handful (less than 10 per sample) matched the PDHK gene and had a length 18–25 nt, the typical range for small RNA. The hits were likely to be degradation products from the PDHK mRNA and other spurious matches. Thus, the construct may have entered a region of the genome that was not hospitable for transcription of the seed specific promoter driving the PDHK inverted repeat, or may have broken up upon entry, or been non-functional for some other unknown reason.

### Phenotypic differences between transgenic plants and non-transgenic segregants

**Fig 2:**
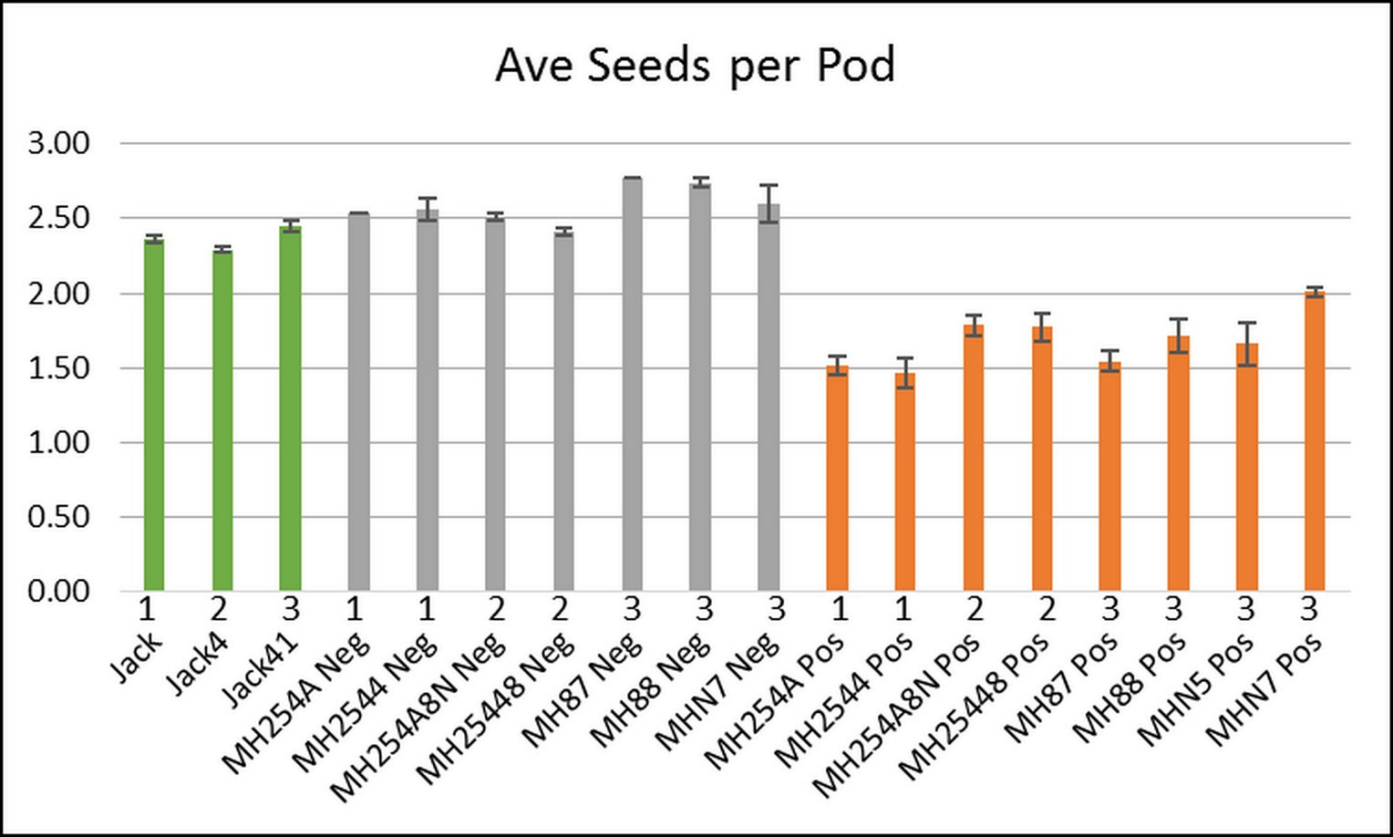
Average seeds per pod at maturity. Each bar represents the average of a group of plants from the same generation and transgenic status (see S2 Table for the number of plants in each group). Green: Jack control plants. Grey: Non-transgenic segregant plants (Neg). Orange: Transformed transgenic (Pos). Numbers above labels indicate generation (1, 2, or 3). Standard error bars shown (standard deviation divided by the square root of N, N being the total number of measurements). The average per group is calculated as the total number of seeds collected for all plants in one group divided by the total number of pods collected for all plants in that group. Averages for each across generations: 2.37 for Jack control (SE = 0.04), 2.59 for non-transgenic segregant (Neg, grey) (SE = 0.05), 1.68 for transformed transgenic (Pos, orange) (SE = 0.06).

**Fig 3:**
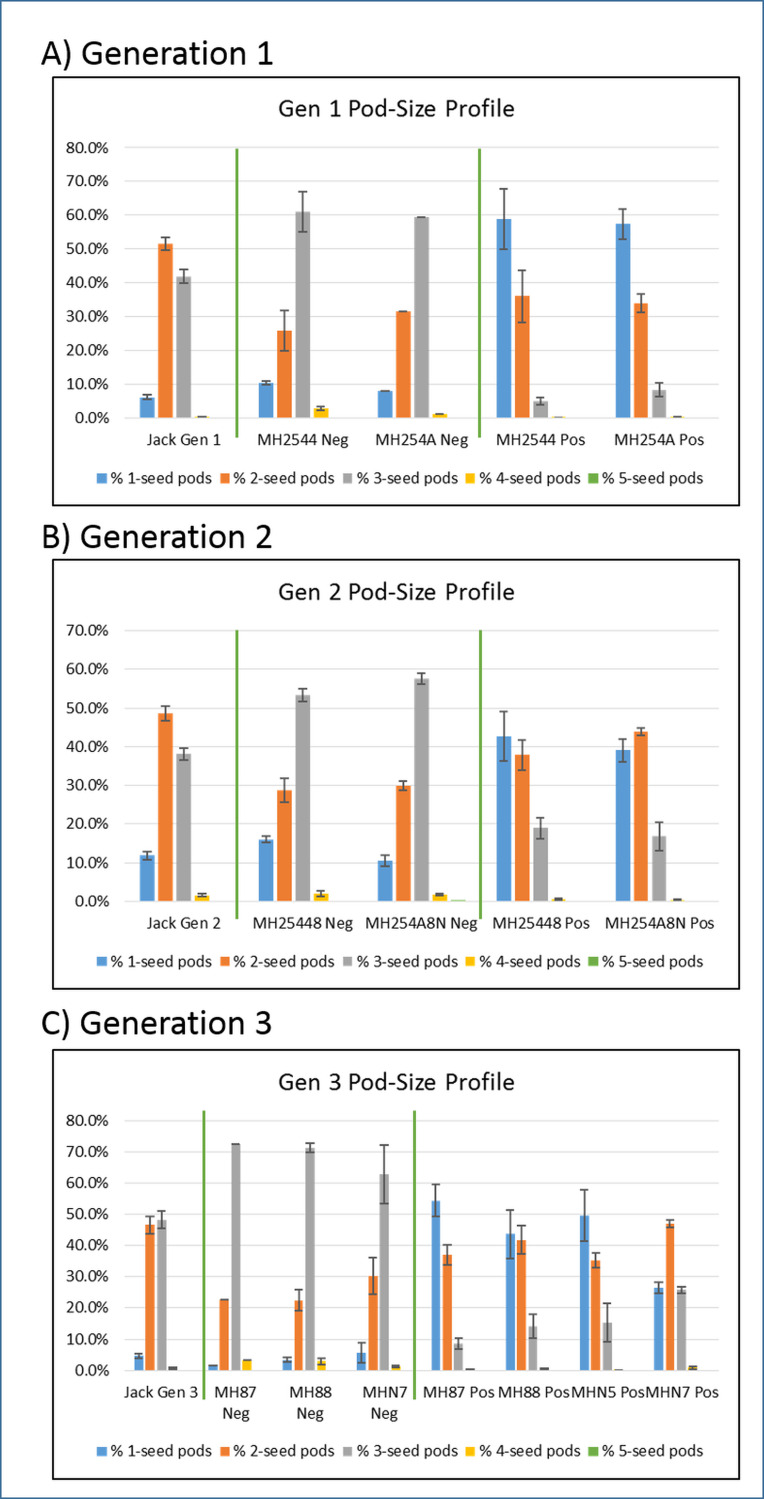
Percentage of pods containing various numbers of seeds, at mature harvest. Panel A: Generation 1. Panel B: Generation 2. Panel C: Generation 3. Jack are non-transformed controls. Neg: Non-transgenic segregant plants. Pos: Transformed transgenic plants. Standard error bars shown (standard deviation divided by the square root of N, N being the total number of measurements). Pods had at least 1 seed and up to 5 seeds each.

**Fig 4:**
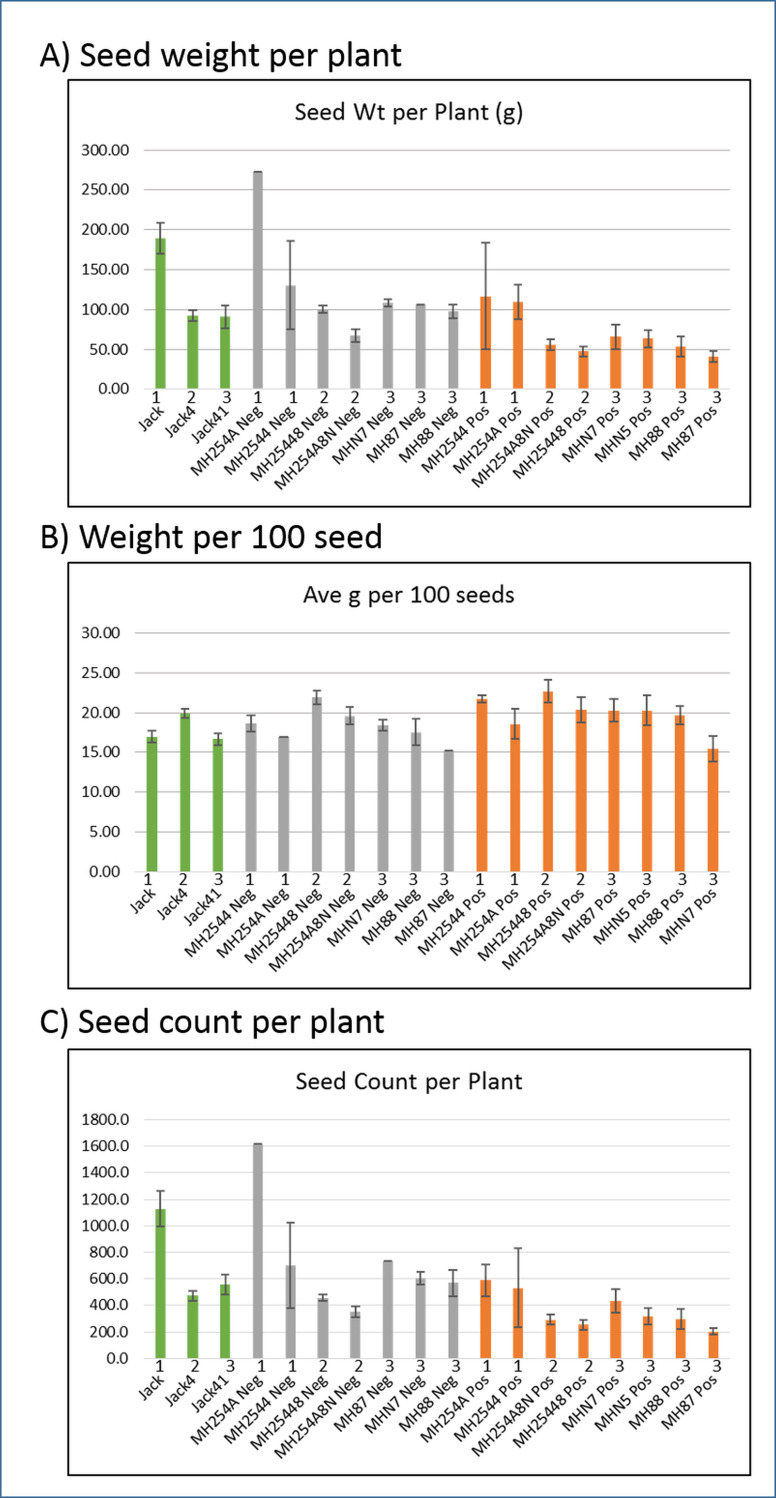
Seed weight and count per plant. Each bar represents the average of a group of plants from the same generation and transgenic status (S2 Table shows the number of plants in each group). Panel A: Weight (g) of all seeds on a plant, averaged. Panel B: Average weight (g) of 100 seeds. Panel C: Number of all seeds on a plant, averaged. Green: Jack control plants. Grey: Non-transgenic segregant plants (Neg). Orange: Transformed transgenic plants (Pos). Numbers above labels indicate generation. Standard error bars shown (standard deviation divided by the square root of N, N being the total number of measurements). Averages for each across generations: A, 124.14g for Jack (SE = 32.6), 126.26g for non-transgenic segregant (SE = 25.5), 69.07g for transgenic (SE = 10.0). B, 17.84g for Jack (SE = 1.0), 18.33g for non-transgenic segregant (SE = 0.79), 19.87g for transgenic (SE = 0.77). C, 718.9 seeds for Jack (SE = 205.6), 719.3 seeds for non-transgenic segregant (SE = 158), 364.5 seeds for transgenic (SE = 48.61). Though the transgenic seeds may be heavier individually, there are fewer of them produced per plant, leading to a net loss in yield.

**Fig 5:**
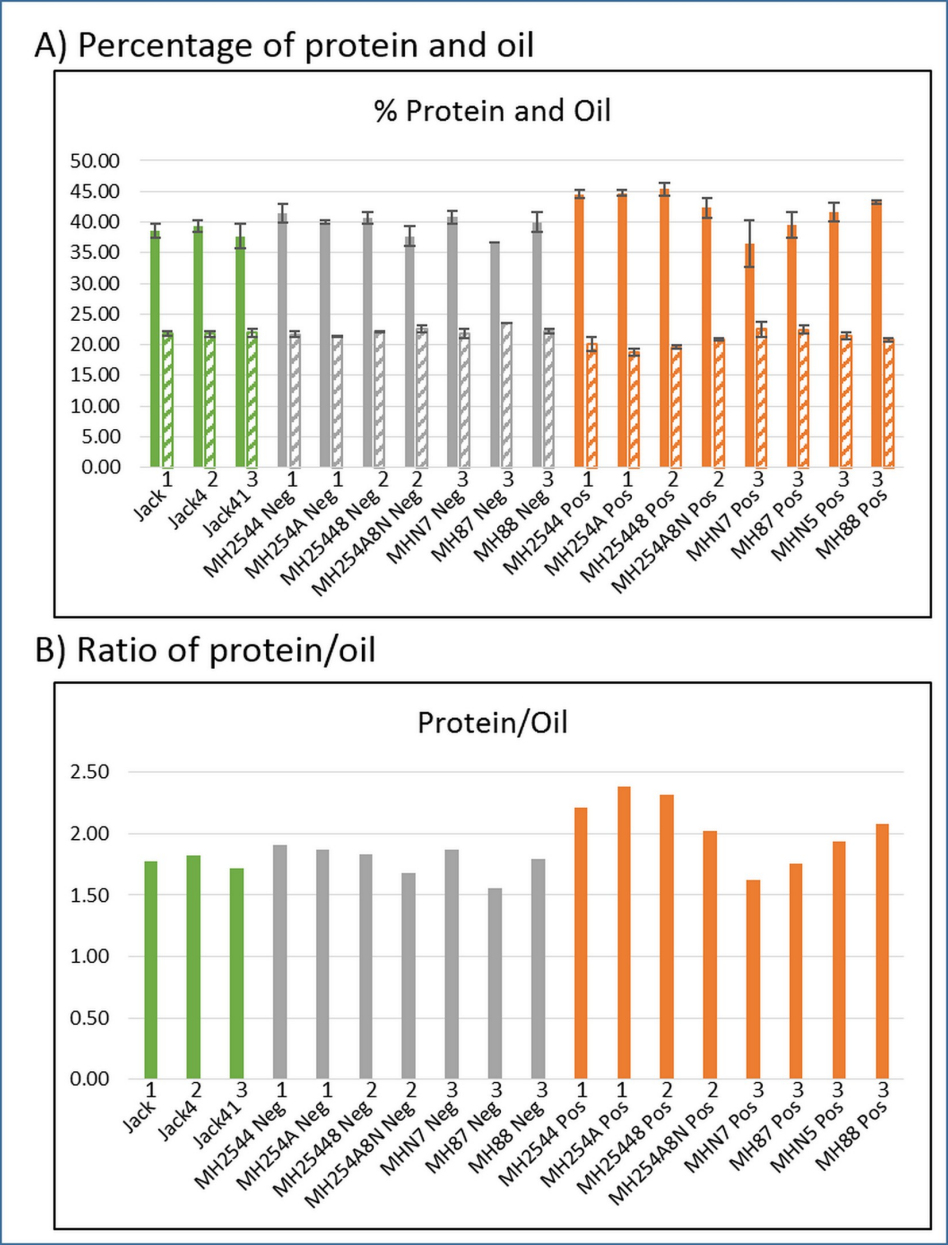
Protein and oil content of seeds. Panel A: Percentage of protein (solid bars) and oil (striped bars) found using near-infrared imaging (NIR). Each bar represents the average of a group of plants from the same generation and transgenic status (S2 Table shows the number of plants in each group). Standard error bars shown (standard deviation divided by the square root of N, N being the total number of measurements). Panel B: Protein/oil ratios calculated from average NIR results. For both panels: green—Jack control plants; grey—non-transgenic segregant plants (Neg); orange—transformed transgenic plants (Pos). Numbers above labels indicate generation. Averages for each across all generations: A, 38.5% protein (SE = 0.48) and 21.9% oil (SE = 0.05) for Jack control, 39.6% protein (SE = 0.66) and 22.2% oil (SE = 0.26) for non-transgenic segregant, 42.2% protein (SE = 1.06) and 20.9% oil (SE = 0.46) for transgenic. B, ratios calculated from the above averages results in 1.76 for Jack, 1.78 for non-transgenic segregant, 2.02 for transgenic.

Seeds from the transgenic plants (as determined by presence of the hygromycin construct) showed significant phenotypic differences from those of the control Jack plants as well as from the non-transgenic segregant plants, as summarized in S2 Table for the three latest generations of plants. S2 Table shows full results for all 79 individual plants as well as for the three groups: the Jack control plants; plants from the transformed lines which were found to be hygromycin positive (Pos); and non-transgenic segregant plants which were found to be negative for hygromycin (Neg). The data from this table are used for the graphs in Figs 2–5. Fig 2 shows the total number of seeds collected from a group of plants, divided by the total number of pods collected from that same group, which gave an estimate of how many seeds were contained in each pod. These data showed distinctions between the control (in green), non-transformed segregants (Neg, in grey), and transgenic (Pos, in orange) plants. The non-transgenic segregants had the highest number of seeds per pod, 2.59 on average (standard error SE = 0.05), while the control Jack plants were slightly lower with an average of 2.37 seeds per pod (SE = 0.04). The transgenic lines were much lower, with an average of 1.68 seeds per pod (SE = 0.06). This is shown another way in Fig 3, which displays the percentage of pods that had a certain number of seeds at maturity. Generally speaking, for soybean pods containing one, two, or three seeds are the most common, with three-seed pods being thought of as “typical.” There were clear differences between the three types in their pod-size profiles. In the Jack control plants, two-seed pods predominated (orange bar), comprising about 50% of the pods at maturity. In the non-transgenic segregants (Neg), 3-seed pods were most commonly found (grey bar), making up at least 50% of the pods at maturity, sometimes over 70%. In the transgenic plants (Pos), one-seed pods were most abundant (blue bar), representing about 40 to 60% of pods. This correlated well with the seeds per pod data; the transgenic plants had far more pods containing just a single seed, so it was expected that they would have fewer seeds per pod on average.

Fig 4A shows the average weight of all seeds per plant with standard errors, by group. The Jack control plants had, on average, about 124 g of seeds per plant. The non-transgenic segregants (Neg) had an almost identical average of 126 g of seeds per plant. However, the transgenic plants (Pos) had roughly half the seed weight as the control and non-transgenic plants, with an average of 69 g of seeds per plant. A lower total seed weight per plant could indicate that the individual seeds are smaller, or that there are fewer per plant. For the transgenic plants (Pos), we found that they produced far fewer seeds than the control and non-transgenic plants. Fig 4B shows the average weight of 100 seeds for each group. The Jack control plants averaged about 17.84 g per 100 seeds (SE = 1.02), with the non-transgenic segregant plants (Neg) just slightly higher at 18.33 g per 100 seeds (SE = 0.79). In the transgenic plants (Pos), the average weight of 100 seeds was 19.87, higher than the Jack control plants (SE = 0.77). However, as shown by Fig 4C, the transgenic plants produced far fewer seeds: each plant produced on average about 719 seeds for Jack control plants and non-transgenic segregant (Neg) plants, compared to 365 seeds per plant for transgenic (Pos) plants. This showed that the lower overall seed weight per plant for the transgenic plants was due to lower seed production compared to the control and non-transgenic segregant plants, although the seeds they did produce tended to be heavier. Overall, this resulted in a net loss of yield for the transgenic plants compared to those without the transgenic insert.

Mature seeds were scanned with a Perten DA7200 near-infrared (NIR) machine to determine the percentage of protein, oil, and other components of agronomic interest. Fig 5 shows the average percentage of protein and oil per plant and Table 1 presents a summary of all of the phenotypic traits including the protein and oil, averaged over all three generations. The Jack control lines contained, on average, 38.5% protein (SE = 0.48) and 21.9% oil (SE = 0.05), leading to a ratio of 1.76. The non-transgenic segregant (Neg) plants had about 1% more protein than the Jack control plants (39.6% SE = 0.66) but only slightly more oil (22.2% SE = 0.26) for a nearly identical average ratio of 1.78. The transgenic (Pos) plants had on average 42.2% protein (SE = 1.06), almost 4% higher than the Jack control plants. Oil was reduced to 20.9% (SE = 0.46), only 1% less than the Jack plants. We found that protein percentage increased, and while oil percentage decreased, it was less than expected proportional to the protein. Therefore the protein/oil ratio of the transgenic plants across all generations was higher with a ratio of 2.02. Other unusual phenotypic traits were observed in the transgenic plants compared to the Jack controls. They took longer to reach maturity (S4 Fig) and produced fewer pods per plants (S5 Fig). Additionally, abnormally undersized cotyledons were occasionally found in immature seeds of the transgenic plants (S6 Fig).

**Table 1:** Averages ± SE of seed data from Figs 2–5 for each of the three groups: Jack controls, Negative segregants lines, or lines Positive (heterozygous) for the hygromycin marker of the transgenic construct.

Group Averages	Jack	Neg	Pos
Fig 2. Seed number per pod	2.37 ±0.04	2.59 ±0.05	1.68 ±0.06
Fig 4A. Seed weight (g) per plant	124.14 ±32.6	126.26 ±25.5	69.07 ±10.0
Fig 4B. Weight (g) per 100 Seed	17.84 ±1.0	18.33 ±0.79	19.87 ±0.77
Fig 4C. Seed number per plant	718.9 ±205.6	719.3 ±158.0	364.5 ±48.61
Fig 5A. Percentage protein	38.5 ±0.48	39.6 ±0.66	42.2 ±1.06
Fig 5A. Percentage oil	21.9 ±0.05	22.2 ±0.26	20.9 ±0.46
Fig 5B. Protein/oil ratios	1.76	1.78	2.02

Standard error is calculated as the standard deviation of the data divided by the square root of n, the number of samples. Protein/oil ratios are simple averages of ratios from Fig 5A.

### Global gene expression differences between transgenic and non-transgenic segregants during two stages of cotyledon development

RNA was extracted from the cotyledons of immature seeds from selected plants at two stages, 10-25mg whole seed fresh weight and 100-200mg whole seed fresh weight (S6 Table). High-throughput next-generation sequencing was performed and the data were aligned to the soybean genome [16] and normalized as RPKM (reads per kilobase of gene model per million mapped reads) [17]. Additionally, the data were analyzed using DESeq [18] to compare the two conditions. Significantly differentially expressed Glyma models were selected based on an adjusted P-value ≤0.05 with a fold change of ≥2 and minimum average RPKM of the over-expressed condition across replicates of ≥5; these models were categorized manually into 29 functional groups (S7–S11 Tables). More gene models were found to be significantly over-expressed in the transgenic (Pos) plants (over 450 gene models) compared to the number of models that were significantly over-expressed in either the non-transgenic segregant (Neg) plants or the Jack control plants (less than 250 gene models). Raw RNA-Seq data is available from the Gene Expression Omnibus, series GSE130483.

**Fig 6:**
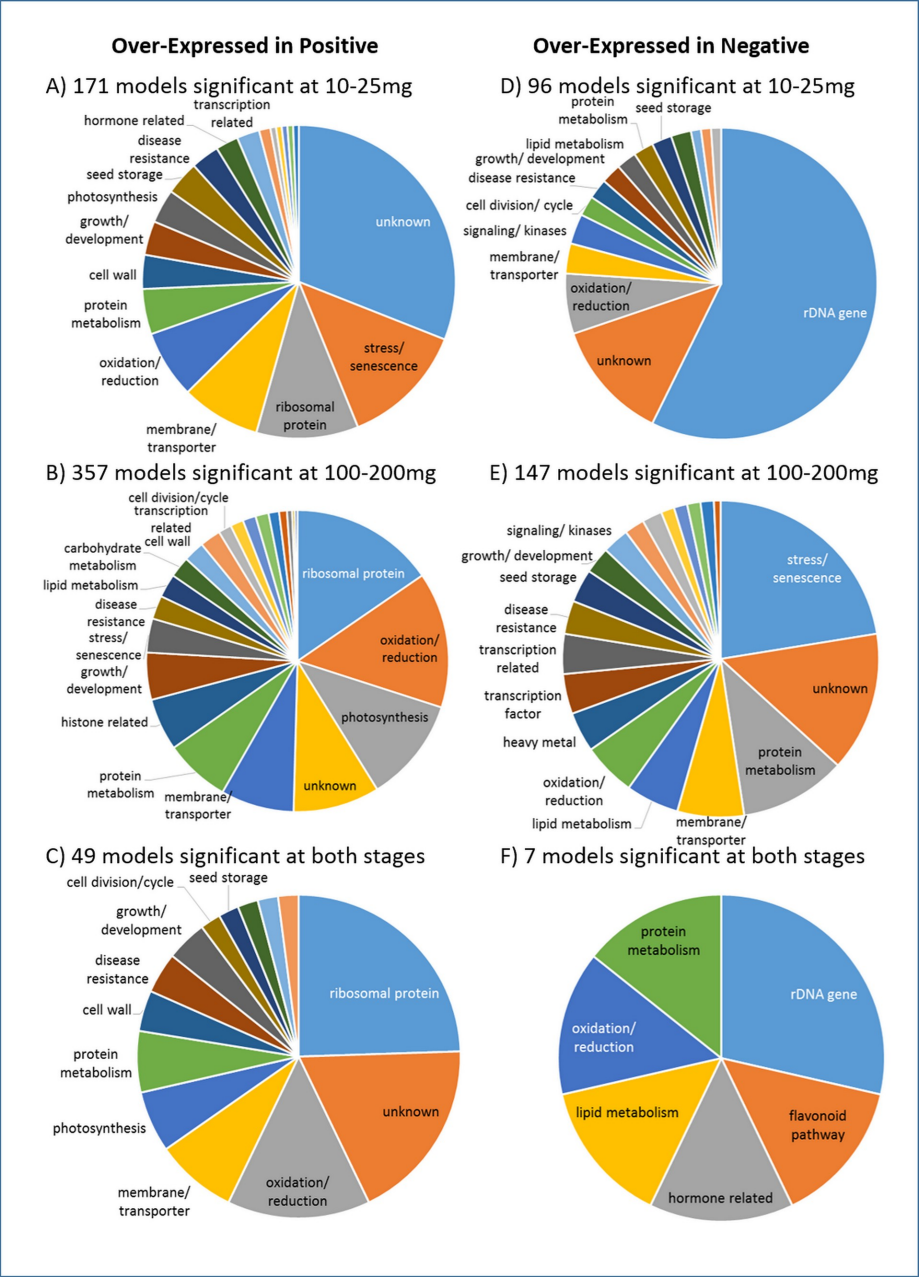
Annotations categorized for genes in transgenic (Positive) plants compared to non-transgenic segregant (Negative) plants. Gene models are significantly differentially expressed in one or both stages of immature cotyledon development (10-25mg or 100-200mg whole seed fresh weight). Pie chart displays the percentage of gene models falling into each functional category. Names of smaller categories have been removed; see S8 Table and S9 Table for full data. A-C: Genes overexpressed in Positive plants compared to Negative plants. D-F: Genes overexpressed in Negative plants compared to Positive plants (underexpressed in Positive). Padj ≤0.05, fold change 2x or greater, average RPKM ≥5 in the overexpressed condition, no splice variants (.1 models only).

Fig 6A–6C and S8 Table show 479 Glyma models which were significantly differentially expressed between the transgenic plants (Pos) and the non-transgenic segregant plants (Neg). A total of 171 of these models were significant at the 10–25 mg stage, 357 models were significant at the 100–200 mg stage, and 49 models were significant at both stages. Both types of plants, transgenic and non-transgenic segregants, were siblings produced by the same transgenic parents, yet the non-transgenic segregant plants were negative for the presence of hygromycin from the transgenic construct. Of the 171 models significant at the younger stage of 10–25 mg, almost one-third (31%) had an unknown function. Another 13% were involved with stress or senescence (e.g., Hsp20/alpha crystalline family, copper/zinc superoxide dismutase) and over 10% were annotated as ribosomal proteins (e.g., 60s acidic ribosomal protein, ribosomal protein S10p/S20e). About 8% had membrane or transporter functions (e.g., major intrinsic protein, lung seven transmembrane receptor). Of the 357 models significant at the older stage of 100–200 mg, those related to ribosomal functions (e.g., ribosomal L15, S25 ribosomal protein) and oxidation and reduction functions (e.g., 2Fe-2S iron-sulfur cluster binding domain, peroxidase) were most common, with about 15% each. About 11% of the gene models had functions related to photosynthesis (e.g., chlorophyll A-B binding protein, photosystem I reaction center subunit VI) and about 9% had unknown functions. Of the 49 models significant at both cotyledon stages, the largest functional category was that of ribosomal-related genes (e.g., 60s acidic ribosomal protein, ribosomal protein S30) with almost 25%. About 18% of the genes had unknown functions, with about 14% in the oxidation and reduction-related category (e.g., glutaredoxin, thioredoxin), and about 8% in the membrane and transporters category (e.g., SecE/Sec61-gamma subunits of protein translocation complex, defender against death family).

A total of 236 Glyma models were significantly over-expressed in the non-transgenic segregant (Neg) plants compared to the transgenic (Pos) plants (Fig 6D–6F and S9 Table). This was about one-half the number that were over-expressed in the transgenic plants and included 55 rDNA (ribosomal DNA) models erroneously identified by the Wm82.a2 genome as gene models; there was only one rDNA model over-expressed in the transgenic plants. Common annotation categories here included genes with unknown functions, stress and senescence genes, and protein metabolism-related functions.

When the transgenic plants were compared to the non-transformed Jack control plants, 463 Glyma models were significant and over-expressed in the transgenics (Pos) (S7 Fig and S10 Table). This was about the same number of genes that were over-expressed in the transgenic plants compared to the non-transgenic segregants. As with the comparison of the transgenic plants (Pos) to the non-transgenic segregants (Neg), the common annotation categories here included ribosomal-related proteins, genes with unknown functions, membrane- and transporter-related proteins, and oxidation and reduction-related genes. A total of 215 Glyma models were significant and over-expressed in the Jack control plants compared to the transgenic (Pos) plants (S7 Fig and S11 Table). This was less than one-half the number that were over-expressed in the transgenic plants and included 77 rDNA models erroneously identified by the Wm82.a2 genome as gene models; there was only 1 rDNA model over-expressed in the transgenic plants. Common annotation categories here included oxidation and reduction-related genes, genes with unknown functions, and signaling and kinase-related genes.

Transcription factors are of particular interest due to their ability to regulate networks of other genes. Only a small percentage of significant genes fell into the transcription factor category, less than 5% across all comparisons (0 to 9 models). However, this analysis imposed an average RPKM of ≥5 in the over-expressed condition; transcription factors can sometimes operate at very low levels. A secondary analysis of the data imposed the same conditions as the first—adjusted p-value ≤0.05, 2x fold change—but with no minimum RPKM. The number of transcription factors increased noticeably, accounting for 3.4% to 9.6% (10 to 95 models) of the significant gene models. Finally, in all comparisons (S8 and S11 Tables), the expression of the PDHK family genes did not vary significantly, as expected since the construct was not effective in expressing the small RNAs. Thus, the phenotypic changes were likely due to insertion of the transgene within a critical region for seed development.

## Discussion

### Alterations in protein and seed size resulting from a transgenic line that induces seed abortion/embryo lethality when homozygous

In the transgenic plants, the developmental program for seeds has obviously been altered. Fewer seeds were produced overall per plant (Fig 2), suggesting they were never formed or were aborted at such an early stage that they could not later be detected in mature pods, leading to more pods classified as containing only one or two seeds instead of the more typical three (Fig 3). As shown by dissections of immature seeds (S6 Fig), even the seeds that appeared to develop may have had abnormal phenotypes such as undersized or missing cotyledons. Fewer pods were formed (S5 Fig), which may have been a direct effect during development or simply the result of all seeds intended for a pod failing to develop. Transgenic plants were also slower to mature than the control plants (S4 Fig). The seeds that did develop on the transgenic plants had a slightly higher average weight (Fig 4), perhaps as a result of receiving a greater proportion of nutrients that otherwise would have been shared with other seeds in the pod. In particular, they had an increased percentage of protein found in the mature seeds, with a disproportionately small loss of oil, leading to an increased protein/oil ratio (Fig 5). However, this increase in weight was not enough to offset the seed losses in these plants, leading to an overall lower total amount of seed weight per plant. Although the transgenic insertion was initially meant to reduce expression of the PDHK gene, ultimately achieving higher seed weights, the expression of this gene had not actually changed. Thus we believe that the effects observed were due to the construct inserting in a gene and interfering with its normal function. A recent study on the effects of biolistic transformation on the broader genome found numerous examples of chromosomal damage, deletions, broken transgenes, and other unintended effects [19].

This transgenic project was unusual in that homozygous lines were not achieved despite the facts that all plants were grown from seeds of confirmed transgenic plants and that soybean is self-fertilizing (S4 Table). One possible explanation is that having two copies of the inserted construct was lethal for the plants, thus homozygotes did not survive beyond an embryonic stage, resulting in a higher rate of aborted seeds in the mutant plants. Heterozygous embryos that received a single intact copy of the gene, and thus some of the functional protein product, were more likely to survive to seed maturation and later successfully germinate, albeit with the developmental consequences described here. In this case, one would expect to see a 2 to 1 ratio of transgenic (heterozygous, with one copy of the disrupted gene) to non-transgenic segregants (homozygous for the normal gene) plants. To attempt to obtain a homozygous line, we initially selected 13 first generation T1 lines that were each positive for the hygromycin gene (by PCR) and planted at least 15 total progeny from each line. However, none of these 13 lines were homozygous for the hygromycin transgene marker. S4 Table shows that over the three most recent generations, there was a ratio of 1.54 to 1 in a population of 61 transformed plants; compared to a 2:1 ratio, this had a χ^2^ test p-value of 0.319, meaning the hypothesis of 2:1 could not be rejected. The typical ratio of 3:1, however, was rejected with a χ^2^ test p-value of 0.0097. While a ratio of 1:1 that would indicate a lethality occurring in the generation of the gametophytes was not rejected by the chi-squared test, that conclusion is not consistent with the formation of some of the partially formed and misshaped embryos as described in S6 Fig. Further microscopic and cytological studies of the developing ovules and young seed at the very early stages a few days after fertilization are needed to determine when the majority of the defects in embryo development appear.

If the embryo could not survive without at least one functional copy of this gene, it was clearly essential for normal development. Embryo-lethal mutations can be challenging to study but there is considerable interest in determining the functions of such genes [20]. In *Arabidopsis*, there are an estimated 750–1000 embryo-defective genes (*EMB*) which, when disturbed, can cause the premature death of the embryo; characteristics of many of these genes are reviewed in [21]. The functions of these genes often overlap with those causing lethal defects in the gametophytes, preventing embryos from forming in the first place, and include such categories as DNA replication, RNA modification, translation of genes from the chloroplast and mitochondria, and fatty acid production. In a recent study of the gene *Vps11* in *Arabidopsis*, which is involved in vacuole formation, [22] researchers were unable to identify homozygous mutants with two copies of the mutated gene; *vps11/+* self-crossed plants had a segregation ratio of 1 to 1.2 of homozygous wild-type plants and heterozygous *vps11/+* mutants. Moreover, the heterozygous plants had a higher percentage of aborted seeds and ovules than the wild-type plants, with some embryos displaying aberrant morphology, including no vacuole. Another recent study in rice found that a gene encoding a subunit of mitochondrial complex I (*OsNDUFA9*), when disturbed by a premature stop codon, produced an aberrant endosperm and no viable embryos in the homozygous mutant [23]. Over 40 embryo-lethal mutants have been induced recently in maize using EMS-treated pollen, following up on older work that induced over 100 lethal mutations in both embryos and endosperms in the hopes of revealing the identities of essential kernel development genes [24, 25].

### Transgenic plants showed increased expression of ribosomal proteins, membrane proteins, and transporters

Examining expression data for over 88,000 gene models across two stages of cotyledon development, with multiple replicates for Jack control plants, transgenic plants, and non-transgenic segregant plants, we saw some notable differences. More gene models were over-expressed (a two-fold or greater change) in the transgenic (Pos) plants compared to the other groups (S7 Table), suggesting that the disruption in the transgenic plants either up-regulated many genes directly (perhaps by interrupting a transcription factor that otherwise kept them down-regulated) or that many genes were indirectly up-regulated in response to the interrupted gene’s lack of function. The transgenic (Pos) and non-transgenic segregant (Neg) plants should have had extremely similar gene expression profiles, as they were derived from the same transgenic parent plants and grown in the same conditions. However, there were 479 gene models which were over-expressed in the transgenic plants compared to their non-transgenic segregant siblings, with about three-quarters of those expressed at the older 100-200mg stage. Looking at the functional categories of these genes, we saw similar kinds of genes recurring as the most common to be over-expressed in the transgenic plants, whether in comparison to the non-transgenic segregant plants or the Jack controls. Genes with unknown functions, for example, were found to be over-expressed in high numbers in the transgenic plants, at both stages and in both comparisons.

Another prominent annotation category over-represented in the positive plants was ribosomal protein-related genes (Fig 6 and S7 Fig). These models were annotated as a variety of ribosomal components (S8 Table and S10 Table), associated with both the large and the small subunits, which stabilize the protein synthesis complex and translate messenger RNA into amino acid chains. Eukaryotes have about 80 ribosomal proteins, which are often represented by multiple duplicate genes for the various subunits, and show high homology to ribosomal proteins from prokaryotes [26]. Numerous studies in plants have found that increased ribosomal protein gene expression is often associated with early embryogenesis [27, 28, 29] and other tissues undergoing increased cell division, such as lateral roots and pollen [30]. In contrast, a recent study in immature soybean cotyledons found that genes encoding ribosomal proteins had very low translational efficiencies, suggesting they are not normally highly expressed at these stages of development [31]. The high amount of ribosomal protein-related genes over-expressed in the transgenic plants could be a further indication of how not just transcription but also translation was increased in these plants, in response to the disruption caused by the transgene vector. Very few ribosomal protein-related genes were over-expressed in either the non-transgenic segregants (Neg) or Jack control plants. Interestingly, gene models labeled as rDNA (ribosomal DNA) showed the opposite pattern, with the vast majority under-expressed in the transgenic plants. This could be an artifact of the RNA extraction process, but nonetheless it was curiously one-sided, with 55 and 77 rDNA models over-expressed in the non-transgenic segregants and Jack control plants, respectively, but only one rDNA model was over-expressed in the transformed plants compared to either line.

Genes with functions related to membrane proteins and transporters were also very commonly over-expressed in the transgenic plants, no matter the stage or which group was being compared (Fig 6 and S7 Fig; S8 and S10 Tables). This is a necessarily broad category, with the main similarity among its members being an association with cell membranes, such as moving substances across them, or directing molecules to them. Eight gene models which were annotated as “major intrinsic proteins” were over-expressed in the transgenic plants compared to both the Jack control plants and the non-transgenic segregant plants (with an additional 2 over-expressed compared with just the non-transgenic segregant plants). No genes with this annotation were over-expressed in the negative or control plants. The major intrinsic proteins, PFAM category PF00230, are protein channels and include aquaporins, known for moving water, ions, and small molecules including small carbohydrates across cell membranes. Thus these transport proteins are crucial for regulating water use in the plant. Some specific major intrinsic proteins have been found to be highly expressed in the developing cotyledons of cotton and peas under normal conditions [32, 33]. As was noted with ribosomal proteins, major intrinsic protein genes have often been found to be up-regulated under conditions of abiotic stress such as salt, chilling, and drought stress in plants including rice, barley, maize, wheat, Arabidopsis, rapeseed, and grapevine [reviewed in 34]. Thus, the increase seen in the transgenic plants may also have been a general reaction to stress caused by the disrupted gene. Other membrane-related gene model annotations found to be over-expressed in the transgenic plants compared to one or both of the other groups included proteins related to the outer (PF08038) or inner (PF02953) mitochondrial membranes or mitochondrial carrier proteins (PF00153), with nine gene models. Seven genes were over-expressed at the 10–25 mg stage in transgenic plants compared to the control, and two additional genes were over-expressed at the 100–200 mg stage in transgenic plants compared to the non-transgenic segregants. In *Arabidopsis*, an over-expressed gene related to the inner mitochondrial membrane increased seedling cotyledon size by enlarging the cells; other genes in the respiratory chain complex, such as those related to ATP synthase, also had increased expression in response to this up-regulated gene [35]. In the current project, three gene models related to ATP synthase (PF00137, PF00231) had higher expression in the transgenic plants compared to the others. The up-regulation of one or more of these mitochondrial membrane-related genes due to the transgenic disruption may have led to the up-regulation of others, possibly with phenotypic effects such as enlarging the immature cotyledons, which were found to weigh slightly more (as whole seeds) than the control and non-transgenic segregant seeds at maturity (Fig 4). The membrane and transporters category was also often under-expressed in the transgenic plants, though with fewer actual gene models compared to those over-expressed. Under-expressed annotations in the transgenic plants included the proton-dependent oligopeptide transporter (POT family, PF00854), dynamin (PF00350), ADP-ribosylation factor (PF00025), vacuolar protein sorting-associated proteins (PF03635), and sulfate transporter family (PF00916).

This split between annotations that were under- and over-expressed in the transgenic plants suggested that the transport of molecules into the cotyledons had not merely been amplified in the transgenic plants, but rather altered in a more nuanced way, with the transport of some substances increasing while that of others decreased. Although fewer seeds develop overall on the transgenic plants (Fig 4), those that did develop tended to be slightly heavier than seeds in the other groups, and to have an altered protein/oil ratio (Fig 5). The plant’s many transporter and membrane proteins certainly play a role in moving these products into the young cotyledons for storage, to be used as food later when the seed germinates. Thus it would seem that the disruption in the transgenic plants somehow perturbed the normal formula of substances to be allocated to the growing cotyledons. Agronomic studies on partial depodding of soybeans (up to 40%) during the rapid grain filling stage have been shown to increase seed size and protein concentration [36, 37], presumably from additional assimilate availability to the remaining seed. No transcriptomics studies have been conducted on this phenonemon. In our investigations, only a small number of immature pods were removed for RNA-Seq sampling from the Jack controls as well as the transgenic and non-transgenic segregants lines. The large number of aborted seed from the embryo lethality was, of course, present in only the transgenic line. The change in seed size and protein in the case of the transgenic embryo lethal may be due partially to the remaining seed receiving an increased share of the assimilate, as well as to a more direct result of gene expression changes set in motion by heterozygosity of the locus interrupted by the transgene. Our transcriptomic data revealed large changes in transcripts for ribosomal proteins, membrane proteins, transporters, and transcription factors between the transgenic and non-transgenic segregants.

### Differentially expressed transcription factors

Transcription factors are extremely important because of their ability to regulate large groups of other genes, potentially causing a cascade of downstream effects if perturbed. If a transcription factor was directly interrupted by the transgene vector, or affected in some way by the interrupted gene, it could have been responsible for many of the phenotypic differences seen in the plants. If a critical transcription factor was interrupted by the insertion event, its expression might be lowered. For the most part, entirely different families of transcription factors were under-expressed in the transgenic plants compared to the other groups. Glyma.20G210500 and Glyma.10G180000 are both annotated as AUX/IAA transcriptional regulators (PF02309); they were under-expressed in the transgenic plants compared to both the control and the non-transgenic segregant plants at multiple stages, or compared to the negative plants at just 100–200 mg, respectively. These are part of the complex network of genes that respond to the plant hormone auxin (indole-3-acetic acid) and affect many aspects of plant development, from roots to floral organs [38]. The products of AUX/IAA transcriptional regulators tend to be short-lived, degrading rapidly as auxin levels increase; however, while active, they can repress other transcription factors, such as the auxin response factors (ARFs) [39]. These genes have also been found to affect fruit size due to cellular expansion [40], embryonic dehydration resistance and long-term seed stability [41, 42], and cotyledon development [43]. Other single transcription factors under-expressed in the transgenic plants included Glyma.05G110700, bHLH (basic helix-loop-helix) transcription factor (PF00010); Glyma.15G173300, CCAAT-binding transcription factor (CBF-B/NF-YA) subunit B (PF02045); and Glyma.01G222600, Tubby transcription factor (PF04525).

In summary, this soybean line contained increased protein and an increased protein/oil ratio, traits which merit further study based on the commercial importance of these products. Because the line never achieved homozygosity, we believe a gene key to normal development was interrupted by a transgenic construct. This resulted in these and other unusual phenotypic and genomic traits, including heavier seed (though fewer per plant) and up-regulation of many genes compared to control lines, such as those coding for ribosomal and transporter-related proteins.

## Materials & methods

### Transgenic construct

The transgenic construct (S1 Fig) was an 8333bp circular plasmid containing hygromycin as selectable markers for plant transformation under control of the 35S promoter and nos terminator regions. The construct also contained 5’ and 3’ lectin segments from soybean; together they flanked a PDHK (pyruvate dehydrogenase kinase) insertion from soybean, which consisted of PDHK Exon 5 in both forward and reverse orientations with Intron 5 (reverse) in between (using Glyma.09G079000). This construct was referred to as PDHK2. The construct sequence was verified by the Center for Computational and Integrative Biology DNA Core at Massachusetts General Hospital (MGH CCIB DNA Core) in Boston, Massachusetts. In S1 Fig the construct was visualized with PlasMapper [44] and its full sequence is given in S1 Data. Raw data from MGH CCIB DNA Core’s sequencing of the construct was deposited at the Short Read Archive, BioProject PRJNA540616. The forward and reverse copies of Exon 5 of PDHK were intended to create a double-stranded RNA segment which would trigger production of small RNAs to degrade all RNA from the PDHK gene, down-regulating it. Lectin is a seed protein that accumulates specifically in the soybean cotyledons during development and the lectin promoter and terminator region have been shown to target foreign genes to the developing cotyledons [45, 46, 47].

### Plant transformation

*Glycine max* cultivar Jack was the control line, and the line which was transformed with the construct. Procedures for soybean somatic embryo initiation, gene gun transformation, selection of transformed tissue, and plant regeneration were carried out as described in [48, 49, 50, 51] with some slight modifications. All early plants were found to be heterozygous for the insertion [15], so lines were propagated from confirmed transgenic plants with high average weight per 100 seeds and higher protein/oil ratios [15]. Plants were grown in the greenhouse under standard conditions.

### Seed harvest and data collection

Immature pods were collected from individual plants in Generations 1 and 2 and shelled. The weight of each seed and its position in the pod (position 1 being closest to where the pod was attached to the plant) were recorded, along with the number of aborted seeds. Seeds were then sorted into various fresh weight ranges. Seeds in selected ranges (10–25 mg or 100–200 mg) were dissected to separate cotyledons from seed coats; normal and abnormal seeds were kept separate. All tissues were flash-frozen in liquid nitrogen then lyophilized. Seeds from all plants were kept separate. At maturity, all pods were collected from individual plants. The pods were shelled and the number of seeds in each pod was recorded. After the seeds had air-dried for about a week, they were weighed in groups of 100, always keeping individual plants separate, and also keeping separate the brown and green seeds from one plant. Each group of 100 seeds was then stored in its own envelope. Any remaining seeds (less than 100) were also weighed and the number of seeds recorded. The average weight per 100 seeds for an individual plant was calculated from the total weight of all seeds (brown and green, normal and abnormal) and the total number of all seeds for a given plant. S2 Table contains complete seed data and standard error calculations.

### DNA extraction

Tissue was collected from each transformed plant and from multiple Jack control plants. Tissue used was a rolled trifoliate, three leaves attached to a short stem, approximately 0.5–1 inches long with the leaves still somewhat rolled up. Tissue was flash-frozen in liquid nitrogen and then lyophilized. DNA was extracted using a procedure based on [52] with minor modifications.

### Primer creation

S3 Table displays primers used. Primers for conventional PCR HygroF2 and HygroR2 were suggested by PrimerQuest (Integrated DNA Technologies), based on the entire sequence of the hygromycin gene (GenBank K01193.1), and manufactured by Integrated DNA Technologies. Digital PCR hygromycin primers chosen by Custom TaqMan Gene Expression Assay (Thermo Fisher) were based on the entire sequence of the hygromycin gene. The forward and reverse primers, and the internal oligo (probe) labeled with FAM-MGB, were manufactured by Thermo Fisher and supplied as a 20x concentration. Digital PCR RAD50 primers were designed using Primer Express, based on a region from the RAD50 gene (Glyma.18G002400.1) determined by BLAST searches to be unique in the soybean genome. The forward and reverse primers, and the internal oligo (probe) labeled with VIC-MGB, were manufactured by Thermo Fisher and used at a 20x concentration of 5 uM for the probe and 18 uM for the primers, in 10 mM Tris pH8 and 1 mM EDTA.

### Conventional PCR and gel electrophoresis

Each 100 ul PCR reaction consisted of 74 ul sterile deionized water; 10 ul 10X PCR Buffer (Invitrogen); 3 ul 50 mM MgCl (Invitrogen); 8 ul 2.5 mM dNTPs (New England Biolabs, N0447S); 1 ul each of 20 uM primers HygroF2 and HygroR2; 2 ul of 0.5ug/ul soybean DNA; and 1ul Taq polymerase (Invitrogen 18038–042). Samples were cycled in 0.2 ml PCR tubes (Corning Axygen, 14-22-262) in PTC-200 PCR machine (MJ Research) with heated lid function. Two control reactions without DNA were run in parallel, one cycling in the PCR machine with the samples and one non-cycling. The PCR program was denaturation for 2 min 20 sec (96°C), annealing 1 min (55°C), and extension 2 min (72°C); repeated for 40 cycles, followed by a final extension of 7 min (72°C) then holding at 15°C until retrieval. A 20 ul aliquot of the reaction with bromophenol dye was analyzed by electrophoresis in a 0.7% agarose gel in 1X Tris-acetate buffer alongside φX174 HaeIII marker (Invitrogen, 15611–015). After approximately 90 min at 150 V, the gel was stained with 10 mg/ml ethidium bromide and imaged with AlphaImagerHP (Alpha Innotech).

### Digital PCR

DNA samples of approximately 0.5 ng/ul were subjected to digital PCR (Keck Center, University of Illinois) with primers for RAD50 (Glyma.18G002400.1) as the single-copy control and for hygromycin as the gene of interest, using the Fluidigm 48.770 Digital Array IFC (Integrated Fluidic Circuit) and standard workflow. Data were analyzed using Fluidigm’s Digital PCR Analysis software, v4.1.2 with quality threshold 0.65, baseline correction linear (derivative), and Target Ct range 15 to 35. The Ct threshold method Auto (Global) was used; this automatically calculated a threshold for each dye and applied it to the entire chip. The threshold for FAM (hygromycin) was 0.004777, threshold for VIC (RAD50) was 0.025692. The key ratio of hygromycin/RAD50 was calculated from the estimated target numbers for each gene [53]. One reaction (for MH254A8N-9) was unsuccessful, but all others worked and confirmed the results from conventional PCR. S1 Table contains ratios and sample number corresponding with Fig 1; example amplification graphs are shown in S2 Fig.

### RNA and small RNA extraction and sequencing

S6 Table contains details of samples sequenced. RNA was extracted using a protocol developed by the Vodkin laboratory, based on [54]. High throughput RNA and small RNA sequencing (RNA-Seq or small RNA-Seq) were performed at the Keck Center (University of Illinois, Urbana, Illinois) using Illumina’s TruSeq Stranded RNA Sample Prep Kit (RNA) or NEB’s NEBNext Small RNA Sample Prep kit (small RNA) per the manufacturer’s instructions. Sequencing was done with Illumina’s HiSeq2500 or HiSeq4000 (RNA) or NovaSeq6000 (small RNA) using the standard Illumina protocol (http://support.illumina.com). A total of 42 to 80 million reads from each library of 100 nt read length were obtained for RNA; for small RNA, a total of 7 to 18 million reads were obtained. Raw data are available from the Gene Expression Omnibus, series GSE130483 for RNA Seq.

### RNA and small RNA alignment and annotation

Alignments of mRNA sequences to all 88,647 Glyma models, including splice variants, from the Williams 82 reference genome of *G*. *max* (Phytozome, Joint Genome Institutes; Wm82.a2.v1) were performed using the Bowtie program v.1 [55] with parameters of 3 mismatches (v3) and 25 alignments (m25). Transcriptome data were normalized in RPKM (reads per kilobase of gene model size per million mapped reads) [17]. The DESeq package [18] was used for statistical testing of total reads aligned, and significantly expressed genes were selected based on an adjusted P-value ≤0.05 controlled for false discovery [56]. Genes with significant P-value between two conditions were further filtered by fold change (2x minimum) and minimum average RPKM across replicates of the over-expressed condition (≥5 RPKM). As shown in S6 Table, there were 3 replicates per stage for the Jack controls; 3 replicates per stage for non-transgenic segregants controls; and 7 replicates per stage for the transgenic plants. Each replicate represents a different plant from either Generation 1 or 2. Glyma model functions were annotated based on PFAM motif annotations provided by Phytozome, and categorized manually into 29 groups based on common function. Before creating the final list of percentages in each category, splice variants were removed, leaving only the primary splice models. Small RNA reads were aligned to the gene PDHK (Glyma.09G079000) using Bowtie with up to 2 mismatches allowed; the list was then filtered to retain sequences between 18–25 nt.

### Near-Infrared Imaging

Near-infrared imaging (NIR) performed using Perten DA7200 using the default settings for soybean. For each plant, seed were divided into envelopes with 100 seed and at least two envelopes of seeds, if available, were scanned. All the values from the seeds of one plant were averaged together to provide the final values for that plant. Approximately ten components of the mature soybean seed were calculated using this technology, including the percent protein and oil as discussed in this paper. Full data are available in S12 Table.

## Supporting information

S1 Fig PDHK2 vector used for transformation. (DOCX)

S2 Fig Example amplification graphs of samples in digital PCR (dPCR). (DOCX)

S3 Fig Conventional PCR results for each plant. (DOCX)

S4 Fig Average days from planting to harvesting. (DOCX)

S5 Fig Pod count per plant. (DOCX)

S6 Fig Normal and abnormal immature seeds. (DOCX)

S7 Fig Annotations categorized for genes in transgenic (Positive) plants compared to non-transgenic jack control plants. (DOCX)

S1 Table Digital PCR (dPCR) ratios that accompany Fig 1. (XLSX)

S2 Table Complete data for individual plants and groups over three generations including standard errors. (XLSX)

S3 Table Primers used in conventional and digital PCR (dPCR). (DOCX)

S4 Table Number of plants in transgenic and non-transgenic segregant categories and chi-squared test results. (XLSX)

S5 Table Transgenic status was confirmed by three different methods for selected plants. (DOCX)

S6 Table Sample information for RNA-Seq, small RNA-Seq, and DESeq data. (XLSX)

S7 Table The number of significantly differentially expressed gene models found by DESeq when comparing transgenic plants, non-transgenic segregants, and jack control plants. (DOCX)

S8 Table Complete DESeq and RNA-Seq data for genes more highly expressed in transgenic (Pos) plants compared to non-transgenic segregant (Neg) plants. (XLSX)

S9 Table Complete DESeq and RNA-Seq data for genes more highly expressed in non-transgenic segregant (Neg) plants compared to transgenic (Pos) plants. (XLSX)

S10 Table Complete DESeq and RNA-Seq data for genes more highly expressed in transgenic (Pos) plants compared to jack (Control) plants. (XLSX)

S11 Table Complete DESeq and RNA-Seq data for genes more highly expressed in jack (Control) plants compared to transgenic (Pos) plants. (XLSX)

S12 Table Near-infrared (NIR) data for mature soybean seeds, individual envelopes. (XLSX)

S1 Data Complete sequence of PDHK2 vector used. (DOCX)

S1 Raw images (PDF)
