# Supplementary material for: An embryo lethal transgenic line manifests global expression changes and elevated protein/oil ratios in heterozygous soybean plants
Source: PLoS One. 2020 Jun 9;15(6):e0233721. doi: 10.1371/journal.pone.0233721 (PMC7282645; doi:10.1371/journal.pone.0233721)
Supplement: S1 Fig — (DOCX) [file pone.0233721.s001.docx]

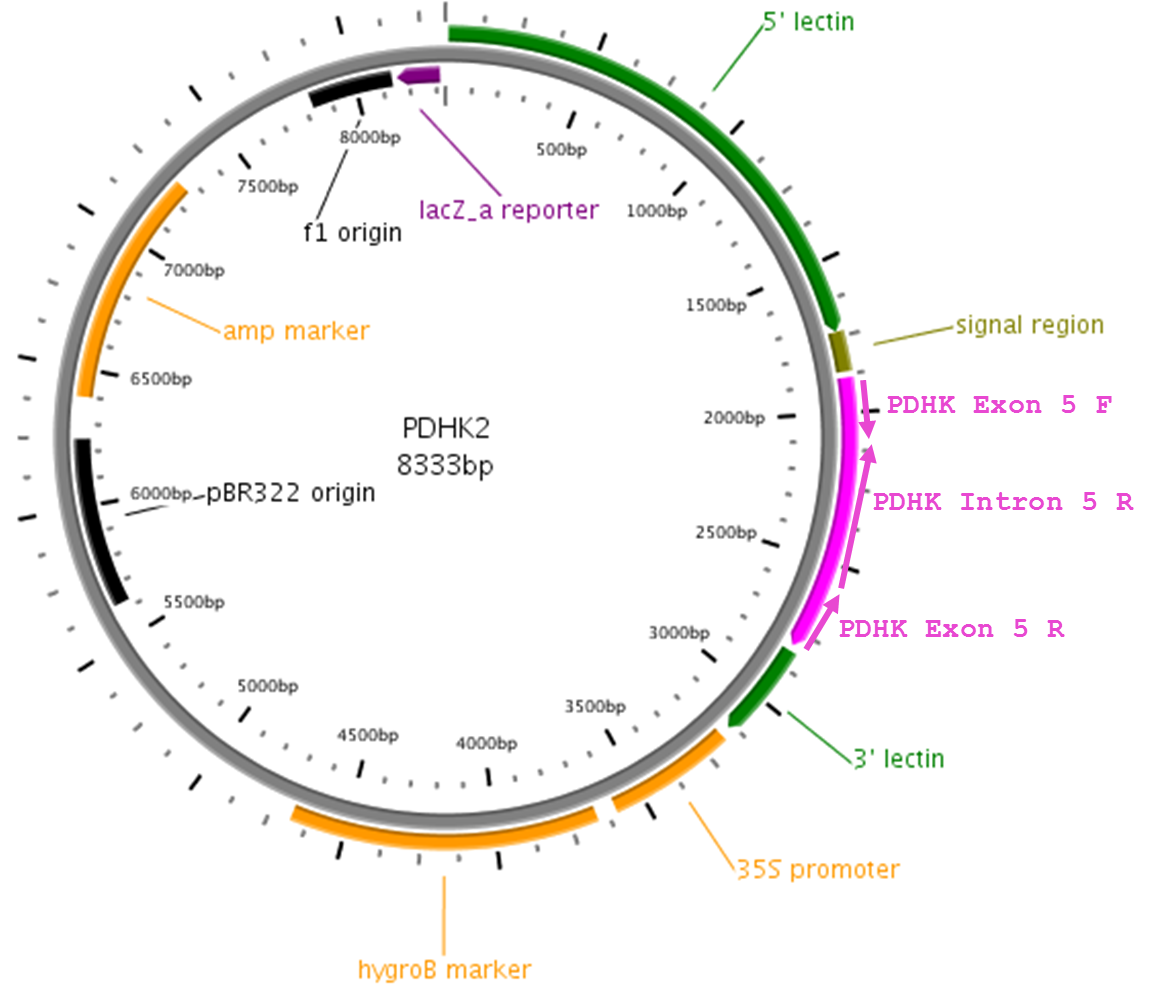


**S1 Figure**. PDHK2 vector used for transformation. It contains Exon 5 of the soybean PDHK (pyruvate dehydrogenase kinase) gene in both forward and reverse orientations, separated by Intron 5 of this gene in the reverse orientation (pink). The vector also contains selectable markers hygromycin and ampicillin (gold) and is contained within sequences of the soybean lectin gene (green). Sequence verified by MGH-Boston, visualized by PlasMapper (Dong et al. 2004). See Appendix S1 for full sequence of construct.
