# Supplementary material for: An embryo lethal transgenic line manifests global expression changes and elevated protein/oil ratios in heterozygous soybean plants
Source: PLoS One. 2020 Jun 9;15(6):e0233721. doi: 10.1371/journal.pone.0233721 (PMC7282645; doi:10.1371/journal.pone.0233721)
Supplement: S2 Fig — (DOCX) [file pone.0233721.s002.docx]

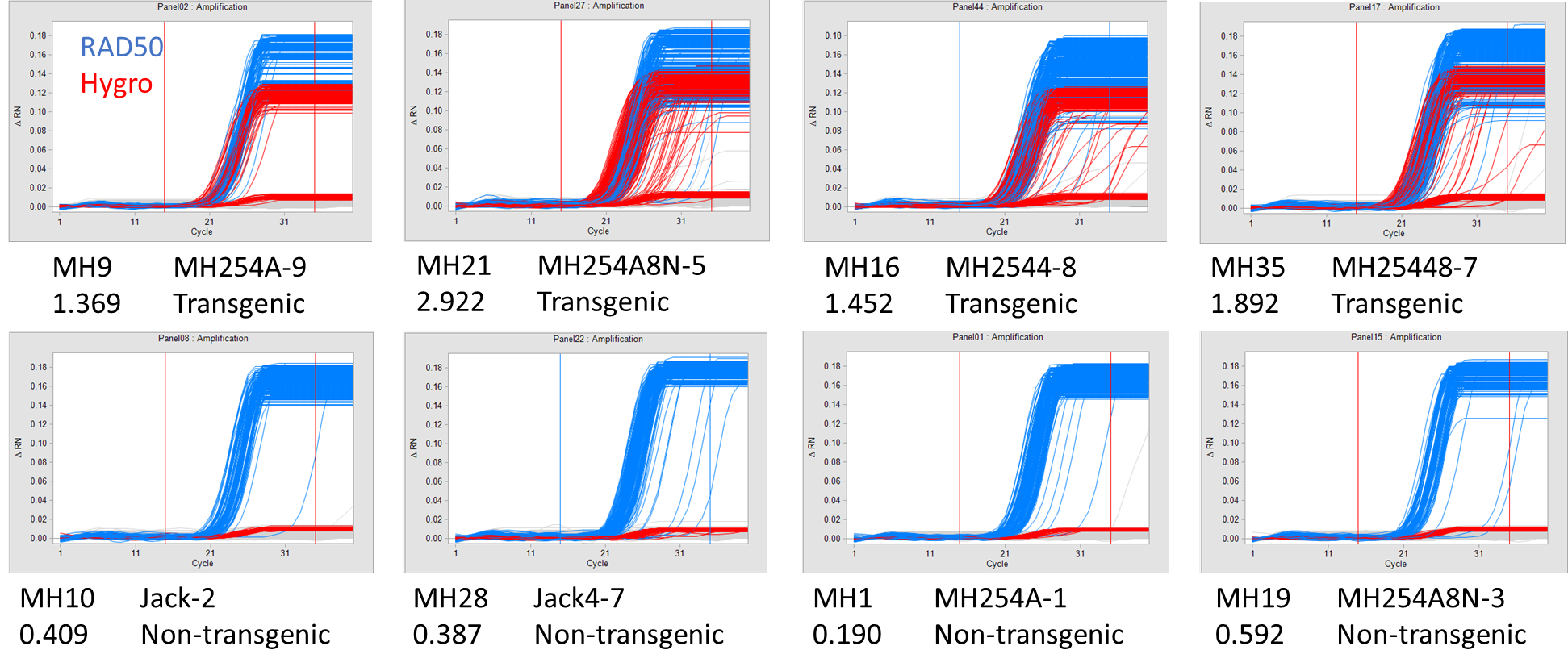


**S2 Figure**. Example amplification graphs of samples in digital PCR (dPCR). All are on the same scale. Blue: RAD50 control gene amplification. Red: hygromycin amplification. Top line, transgenic samples. Bottom line, non-transgenic segregant samples. Ratio of hygromycin/RAD50 given below each graph, along with sample number and plant. Generation 1: MH9, MH16, MH10 (R205/R207 RNA-Seq data), MH1 (R239/R240 RNA-Seq data). Generation 2: MH21 (R223/R229 RNA-Seq data), MH35 (R225/R231 RNA-Seq data), MH28, MH19 (R244 RNA-Seq data). Vertical lines show target Ct range (cycles 15 to 35). In the transgenic samples (top line) the red hygromycin amplifies as does the blue RAD50, resulting in a ratio over 1. In the non-transgenic segregant samples (bottom line), the red hygromycin remains flat while the blue RAD50 amplifies, resulting in a ratio less than 1. Final ratios as shown here and plotted in Figure 1 are calculated from the estimated target numbers for each gene as described in the Methods section; all ratios are listed in Table S1.
