# Supplementary material for: An embryo lethal transgenic line manifests global expression changes and elevated protein/oil ratios in heterozygous soybean plants
Source: PLoS One. 2020 Jun 9;15(6):e0233721. doi: 10.1371/journal.pone.0233721 (PMC7282645; doi:10.1371/journal.pone.0233721)
Supplement: S3 Fig — (DOCX) [file pone.0233721.s003.docx]

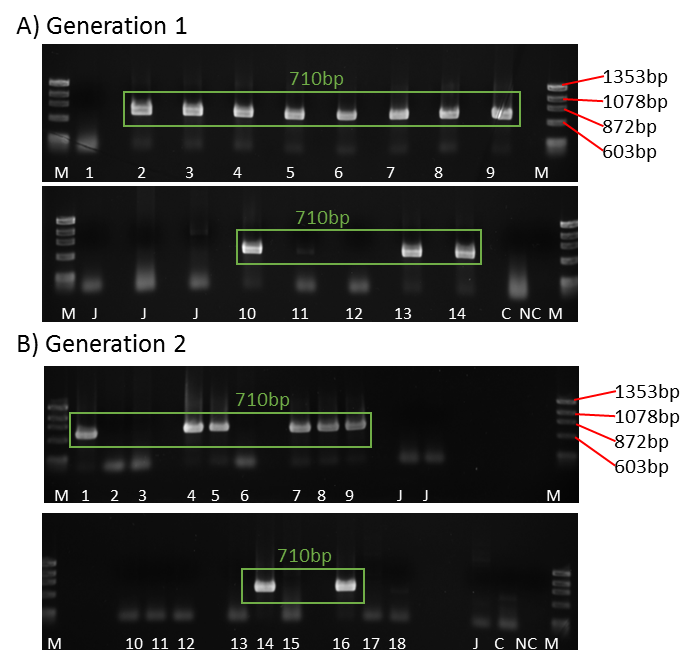


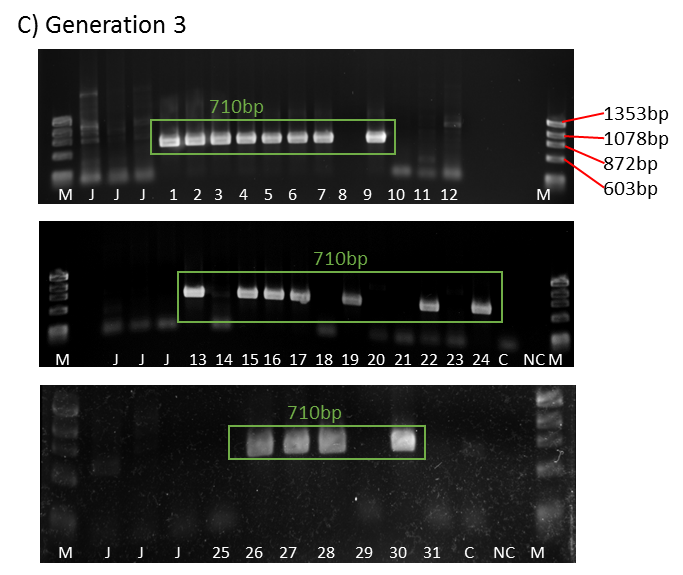


**S3 Figure**. Conventional PCR results for each plant. A PCR amplicon of 710bp (e.g., Panel A Lane 2) indicates the plant was positive for the hygromycin resistance gene on the transgenic construct. M, marker: φX174 Hae III DNA fragments. J: Jack control plants for each generation. C: Cycling control. NC: Non-cycling control. S2 Table contains full details about each plant.

Panel A, Generation 1 plants.

Lanes 1-9: MH254A plants 1-9.

Lanes 10-13: MH2544 plants 1, 4, 7, 8.

Lane 14: MH2333 plant 1 (not discussed here).

Panel B, Generation 2 plants.

Lanes 1-9: MH25448 plants 1-9.

Lanes 10-18: MH254A8N plants 1-9.

Panel C, Generation 3 plants.

Lanes 1-6: MHN5 plants 1-6.

Lanes 7-12: MH88 plants 1-6.

Lanes 13-18: MHN7 plants 1, 2, 3, 5, 7, 8.

Lanes 19-24: MH87 plants 1-6.

Lane 25: MHN7 plant 9.

Lanes 26, 27: MH87 plants 7, 9.

Lanes 28-31: MH88 plants 7-9, 2. MH88 plant 2 was repeated in Lane 31 as the first reaction (Lane 8) did not work.

Plant Gel image RNA-Seq # Transgenic status

MH254A-1 Panel A, Lane 1 R239/R240 Negative

MH254A-4 Panel A, Lane 4 R221/R227 Positive

MH254A-6 Panel A, Lane 6 R228 Positive

MH254A-8 Panel A, Lane 8 R222 Positive

MH2544-1 Panel A, Lane 10 R209/R211 Positive

MH2544-4 Panel A, Lane 11 R210/R212 Negative

MH25448-7 Panel B, Lane 7 R225/R231 Positive

MH25448-8 Panel B, Lane 8 R226/R232 Positive

MH254A8N-1 Panel B, Lane 10 R243 Negative

MH254A8N-3 Panel B, Lane 12 R244 Negative

MH254A8N-5 Panel B, Lane 14 R223/R229 Positive

MH254A8N-7 Panel B, Lane 16 R224/R230 Positive

Selected plants, both positive and negative, were subjected to RNA-Seq at one or two different stages (R#). See S6 Table for further details.
