# Supplementary material for: An embryo lethal transgenic line manifests global expression changes and elevated protein/oil ratios in heterozygous soybean plants
Source: PLoS One. 2020 Jun 9;15(6):e0233721. doi: 10.1371/journal.pone.0233721 (PMC7282645; doi:10.1371/journal.pone.0233721)
Supplement: S5 Fig — (DOCX) [file pone.0233721.s005.docx]

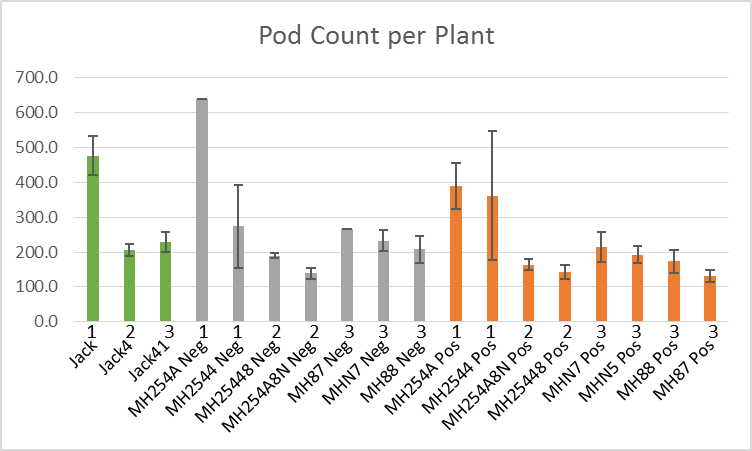


**S5 Figure**. Pod count per plant. Each bar represents the average of a group of plants from the same generation and transgenic status (Table 1 shows the number of plants in each group). Standard error bars shown (standard deviation divided by the square root of N, N being the total number of measurements). Green: Jack control plants. Grey: Non-transgenic segregant plants (Neg). Orange: Transformed transgenic plants (Pos). Numbers above labels indicate generation. Averages for each across all generations: 303.3 pods for Jack control (standard error 86.7), 278.0 pods for non-transgenic segregant (standard error 62.4), 221.3 pods for transgenic (standard error 34.9).
