# Supplementary material for: An embryo lethal transgenic line manifests global expression changes and elevated protein/oil ratios in heterozygous soybean plants
Source: PLoS One. 2020 Jun 9;15(6):e0233721. doi: 10.1371/journal.pone.0233721 (PMC7282645; doi:10.1371/journal.pone.0233721)
Supplement: S6 Fig — (DOCX) [file pone.0233721.s006.docx]

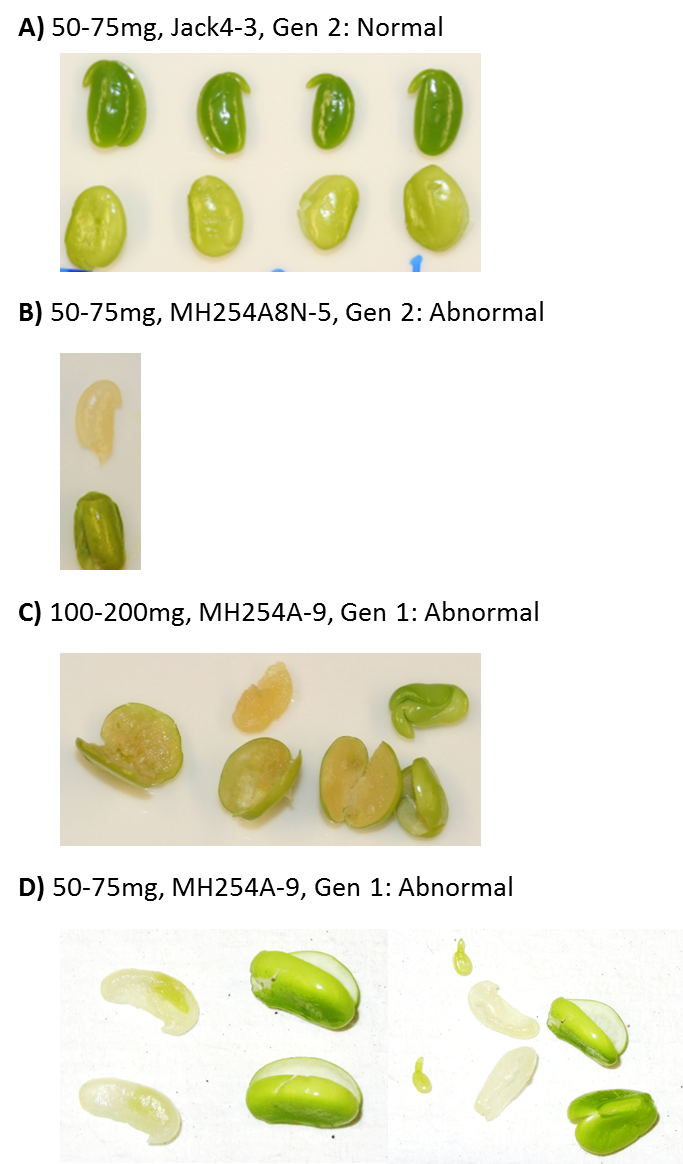


**S6 Figure**. Normal and abnormal immature seeds. A: Jack control line, normal. B: Transformed transgenic plant, abnormal (lab RNA-Seq # R223/R229). A translucent cotyledon-shaped substance was found inside the seed coat, instead of the green cotyledons and embryo. C: Transformed transgenic plant, abnormal. The seed coat was filled with a thick tan substance instead of cotyledons and embryo. Also, double embryos were found inside one seed coat. D: Transformed transgenic plant, abnormal. A thick white substance containing underdeveloped cotyledons with disproportionately large embryo was found inside the seed coats. Brightness and contrast adjusted for clarity.
