# Supplementary material for: An embryo lethal transgenic line manifests global expression changes and elevated protein/oil ratios in heterozygous soybean plants
Source: PLoS One. 2020 Jun 9;15(6):e0233721. doi: 10.1371/journal.pone.0233721 (PMC7282645; doi:10.1371/journal.pone.0233721)
Supplement: S7 Fig — (DOCX) [file pone.0233721.s007.docx]

**
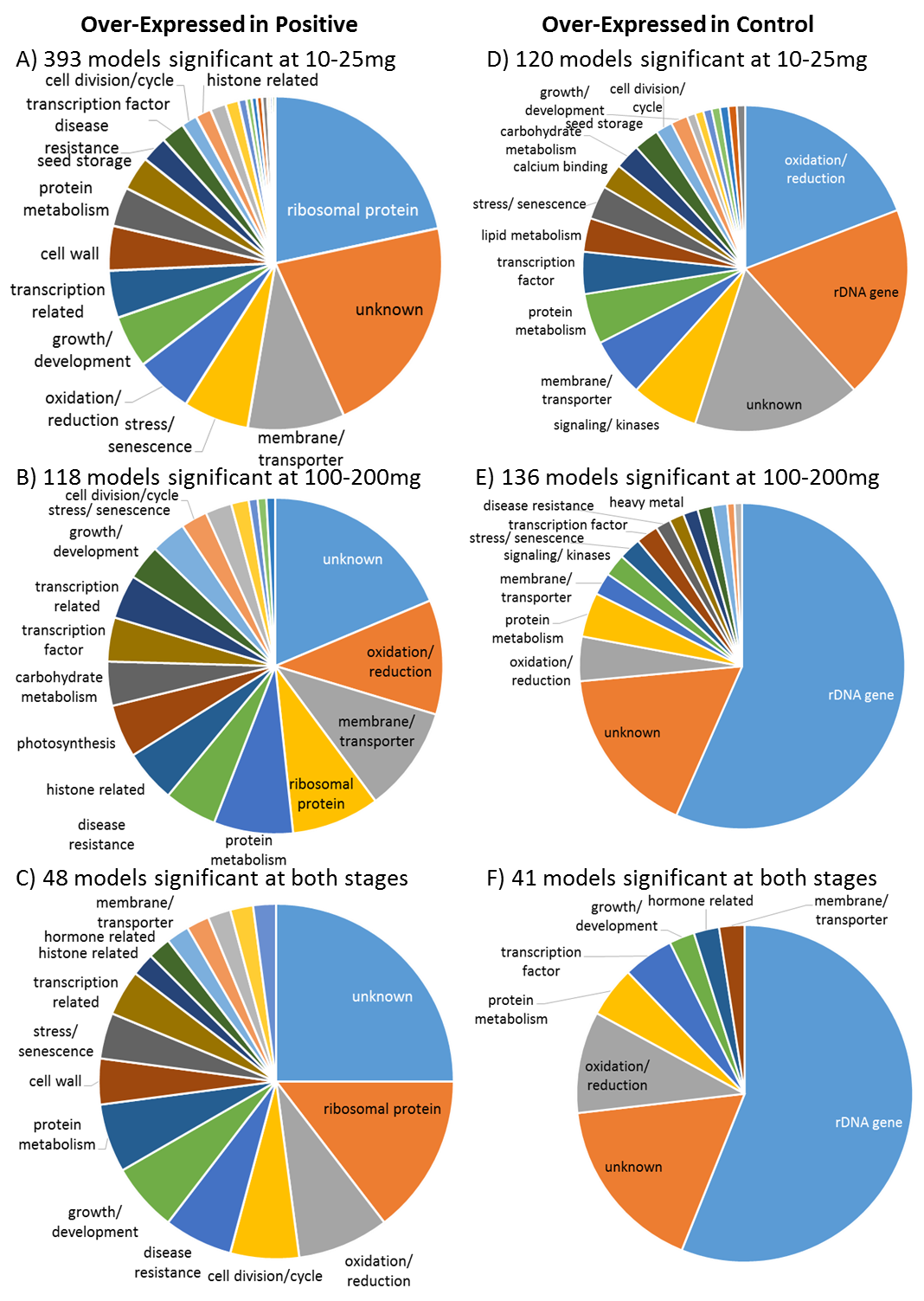
**

**S7 Figure**. Annotations categorized for genes in transgenic (Positive) plants compared to non-transgenic Jack control plants. Gene models are significantly differentially expressed in one or both stages of immature cotyledon development (10-25mg or 100-200mg whole seed fresh weight). Pie charts display the percentage of gene models falling into each functional category. Names of smaller categories have been removed; see Tables S10 and S11 for full data. A-C: Genes overexpressed in Positive plants compared to control plants. D-F: Genes overexpressed in control plants compared to Positive plants (underexpressed in Positive). Padj ≤0.05, fold change 2x or greater, average RPKM ≥5 in the overexpressed condition, no splice variants (.1 models only).
