# Supplementary material for: An embryo lethal transgenic line manifests global expression changes and elevated protein/oil ratios in heterozygous soybean plants
Source: PLoS One. 2020 Jun 9;15(6):e0233721. doi: 10.1371/journal.pone.0233721 (PMC7282645; doi:10.1371/journal.pone.0233721)
Supplement: S3 Table — (DOCX) [file pone.0233721.s010.docx]

**S3 Table**. Primers used in conventional and digital PCR (dPCR).

| Sequence | Name | Type | Amplicon Length (nt) | Purpose | Target |
| --- | --- | --- | --- | --- | --- |
| GGATATGTCCTGCGGGTAAA | HygroF2 | Forward | 710 | Conventional PCR | Hygromycin resistance gene on transgenic construct |
| CCGTCAACCAAGCTCTGATA | HygroR2 | Reverse |  |  |  |
| TCTCGATGAGCTGATGCTTTGG | N/A | Forward | 82 | Digital PCR | Hygromycin resistance gene on transgenic construct |
| ACTGCCCCGAAGTCC | N/A | Internal oligo labeled with FAM-MGB |  |  |  |
| GGATTTCGGCTCCAACAATGTC | N/A | Reverse |  |  |  |
| GCTACCAGTTCAGCTGGACATATG | RADFor | Forward | 73 | Digital PCR | Glyma.18G002400.1 (RAD50 DNA-repair protein) as single-copy control gene in soybean |
| TCTCGCCGTAGAATC | RADdye | Internal oligo labeled with VIC-MGB |  |  |  |
| ATTGCTGAAAATGAGACTCTGCAT | RADRev | Reverse |  |  |  |

Reverse: reverse complement sequence given.
