# Supplementary material for: An embryo lethal transgenic line manifests global expression changes and elevated protein/oil ratios in heterozygous soybean plants
Source: PLoS One. 2020 Jun 9;15(6):e0233721. doi: 10.1371/journal.pone.0233721 (PMC7282645; doi:10.1371/journal.pone.0233721)
Supplement: S5 Table — (DOCX) [file pone.0233721.s012.docx]

**S5 Table**. Transgenic status was confirmed by three different methods for selected plants.

| Plant | Conventional PCR | dPCR | Hygromycin Resistance Gene RNA-Seq | RNA-Seq # |
| --- | --- | --- | --- | --- |
| MH254A-4 | Positive | Positive | Positive | R221/R227 |
| MH254A-6 | Positive | Positive | Positive | R228 |
| MH254A-8 | Positive | Positive | Positive | R222 |
| Jack-2 | Negative | Negative | Negative | R205/R207 |
| Jack-4 | Negative | Negative | Negative | R206/R208 |
| MH2544-1 | Positive | Positive | Positive | R209/R211 |
| MH2544-4 | Weak positive | Negative | Negative | R210/R212 |
| MH254A8N-5 | Positive | Positive | Positive | R223/R229 |
| MH254A8N-7 | Positive | Positive | Positive | R224/R230 |
| MH25448-7 | Positive | Positive | Positive | R225/R231 |
| MH25448-8 | Positive | Positive | Positive | R226/R232 |

Of 79 plants in the experiment, 67 were subjected to conventional PCR (excepting some of the Jack controls in generations 1 and 2). 37 plants in Generations 1 and 2 (again excepting some of the Jack controls) were subjected to digital PCR (dPCR). Finally, 11 plants in Generations 1 and 2 (all shown above) were subjected to high-throughput RNA-Seq (immature cotyledons from whole seeds at 10-25mg and/or 100-200mg fresh weight; R# indicated), with the results searched for reads matching the hygromycin resistance gene. Positive indicates that the hygromycin resistance gene was detected with either primers (conventional PCR or dPCR) or matching RNA-Seq reads; negative indicates it was not detected. One “weak positive” plant by conventional PCR was definitively confirmed as negative with other methods. For all other plants, all methods used agree completely.
