# Supplementary material for: An embryo lethal transgenic line manifests global expression changes and elevated protein/oil ratios in heterozygous soybean plants
Source: PLoS One. 2020 Jun 9;15(6):e0233721. doi: 10.1371/journal.pone.0233721 (PMC7282645; doi:10.1371/journal.pone.0233721)
Supplement: S7 Table — (DOCX) [file pone.0233721.s014.docx]

**S7 Table**. The number of significantly differentially expressed gene models found by DESeq when comparing transgenic plants, non-transgenic segregants, and Jack control plants.

| List name | Conditions | Total gene models | Gene models sig at 10-25mg | Gene models sig at 100-200mg | Gene models sig at both stages |
| --- | --- | --- | --- | --- | --- |
| Pos vs Neg, higher in Pos | A | 479 | 171 | 357 | 49 |
| Pos vs Ctrl, higher in Pos | A | 463 | 393 | 118 | 48 |
| Pos vs Neg, higher in Neg | B | 236 | 96 | 147 | 7 |
| Pos vs Ctrl, higher in Ctrl | C | 215 | 120 | 136 | 41 |

Pos is transgenic; Neg is non-transgenic segregants; Ctrl is Jack control plants. The gene models are significant at one (10-25mg, 100-200mg) or both stages of immature cotyledon development studied. The adjusted p-value at ≤0.05 was used (Padj) in all comparisons; only .1 models used (no splice variants). RPKM, reads per kilobase of gene model size per million mapped reads. See S6 Table for more details of DESeq comparisons.

Conditions:

A) Fold change ≥2 (higher in transgenic) and average RPKM ≥5 across 7 transgenic replicates versus 3 non-transgenic segregants or Jack controls

B) Fold change ≤0.5 (higher in non-transgenic segregants) and average RPKM ≥5 across 3 non-transgenic segregant replicates versus 7 transgenic replicates

C) Fold change ≤0.5 (higher in control) and average RPKM ≥5 across 3 Jack control replicates versus 7 transgenic replicates
