# Supplementary material for: An embryo lethal transgenic line manifests global expression changes and elevated protein/oil ratios in heterozygous soybean plants
Source: PLoS One. 2020 Jun 9;15(6):e0233721. doi: 10.1371/journal.pone.0233721 (PMC7282645; doi:10.1371/journal.pone.0233721)
Supplement: S1 Data — (DOCX) [file pone.0233721.s020.docx]

**S1 Data**. Complete sequence of PDHK2 vector used.

Full 8333nt sequence of the PDHK2 construct used in this project. This is the reverse complement of the sequence returned by MGH-Boston, rearranged to start with lectin.

GGGCGAATTCTCTAGAAAAGTTAACCCTTCGAAGATGATACTGACATTAACACCATTTTTTAATATTGTTTTTCTATATCGTTATTGATCTCAGCACATTCTTAGAAAGATATTTAAATTAGATAAAAGTAAATTTATATATATATATATATATATATATATATATATATATATATATATAAATGTAACATAAATCTATGGTCAATTACAATATTTAATTAAATAAAATAGAAATATAAACACCACTTTAATTTGACTCGGATACATGCATCCATAAAGACTACAAAAGGCAAAAAGAGAAGGAAATGAGATACGAATATATGTCATAAGTATATATAGGTGACAAGGGCAAATTAAATAGGTTGGTATTTAAATGCAAAATCCTATGTTTGATAAAGAATGGTATGAAAAACAGGCAAAGTTAATTGCAATTCAAAGGTGAACAAAGCATTTCTTTGTCTACACTAATGGCATGTCTAAGTAAATTATTAGTCTTGTATCTATATGTCCACAAGTTATTAATTAGTCTTATACTATCAAAAACAAGTTAAGTTGCAAATCAAACATGAACAAAGCATTTGTGTTGTAACCTACGAAAAAATACCCTAACATACTGATACGAATAATGTGGCCTAAATTGATCGTTTACCAAATTACGGTGCTGGAAAAAAAAATTGCTCCTTTACCAACAAAATTAAGAACTGATACATCTTGTTTTTTGTCACTGAAGATAAACACGTGATCTTTGGCAAAACATAAAGGCCAACAAAACAAACTTGTCTCATCCCTGAATGATTCGAATGCCATCGTATGCGTGTCACAAAGTGGAATACAGCAATGAACAAATGCTATCCTCTTGAGAAAAGTGAATGCAGCAGCAGCAGCAGACTAGAGTGCTACAAATGCTTATCCTCTTGAGAAAAGTGAATGCAGCGGCAGCAGACCTGAGTGCTATATACAATTAGACACAGGGTCTATTAATTGAAATTGTCTTATTATTAAATATTTCGTTTTATATTAATTTTTTAAATTTTAATTAAATTTATATATATTATATTTAAGACAGATATATTTATTTGTGATTATAAATGTGTCACTTTTTCTTTTAGTCCATGTATTCTTCTATTTTTTCAATTTAACTTTTTATTTTTATTTTTAAGTCACTCTTGATCAAGAAAACATTGTTGACATAAAACTATTAACATAAAATTATGTTAACATGTGATAACATCATATTTTACTAATATAACGTCGCATTTTAACGTTTTTTTAACAAATATCGACTGTAAGAGTAAAAATGAAATGTTTGAAAAGGTTAATTGCATACTAACTATTTTTTTTCCTATAAGTAATCTTTTTTGGGATCAATTGTATATCATTGAGATACGATATTAAATATGGGTACCTTTTCACAAAACCTAACCCTTGTTAGTCAAACCACACATAAGAGAGGATGGATTTAAACCAGTCAGCACCGTAAGTATATAGTGAAGAAGGCTGATAACACACTCTATTATTGTTAGTACGTACGTATTTCCTTTTTTGTTTAGTTTTTGAATTTAATTAATTAAAATATATATGCTAACAACATTAAATTTTAAATTTACGTCTAATTATATATTGTGATGTATAATAAATTGTCAACCTTTAAAAATTATAAAAGAAATATTAATTTTGATAAACAACTTTTGAAAAGTACCCAATAATGCTAGTATAAATAGGGGCATGACTCCCCATGCATCACAGTGCAATTTAGCTGAAGCAAAGCAATGGCTACTTCAAAGTTGAAAACCCAGAATGTGGTTGTATCTCTCTCCCTAACCTTAACCTTGGTACTGGTGCTACTGACCAGCAAGGCAAACTCAGCGGCCGCGAATTCACTAGTATTGCAAGAAGTGGTTTGCCTAAAATTTTTACATATCTATATAGTACTGCCAGAAACCCATTGGATGAGCATTCGGATCTTGGAATAGGTGATAATGTGACAATGGCTGGATATGGATATGGTCTTCCTATTAGTCGTCTATATGCTCGGTATTTTGGAGGTGAACTAGTGATTCCATGACTGCATCAGAAAGCTTTATTGTTGACTTTGAAGCAAGCAAAAGTATATTGTAAAGAGGAGCAGTATAAAAAAAAAAAAAGAACAACAAACCAACACTATAAAATGTAATTAGATATCTATATATACAAATCAACTCTTCAAGTTTGGGGTATTGAAGTGCTCAGCAGGCTTTAGAAAATCCGAAAAACCACCCCAGAAAGTATGAGCTTACAAAGCTCTAATGTGTAATGAGTAATGTCTAACTTACACATGGGAGAAAATAATAATATTCACTTGATACTTCAATGAAAGCAATAATTATTATTGTAGAAGTTCAAATGGATCTCAAAGACATGTAACCAGAAAATAAGGTTGATACAGTTTCATCATATCAAGTGCAATTAGGTCTCATATCTCAAAACTCCTATATTCTAAATGGAGAAGATACAAAGAGTTGTAACTCTTTTAAGTGCTAAAGTAGATAGAATTACAAAAGGTGGAAGTGTATCTATCAGAAAAATATTATAAATGAAGGGGAAAACAGGTAATAGCCTACTCACCATATCCTTCCATAGAGATTATTTGAAGATCACCTCCAAAATACCGAGCATATAGACGACTAATAGGAAGACCATATCCATATCCAGCCATTGTCACATTATCACCTATTCCAAGATCCGAATGCTCATCCAATGGGTTTCTGGCAGTACTATATAGATATGTAAAAATTTTAGGCAAACCACTTCTTGCAATATCGAATTCCCGCGGCCGCCATGTGACAGATCGAAGGAAGAAAGTGTAATAAGACGACTCTCACTACTCGATCGCTAGTGATTGTCATTGTTATATATAATAATGTTATCTTTCACAACTTATCGTAATGCATGTGAAACTATAACACATTAATCCTACTTGTCATATGATAACACTCTCCCCATTTAAAACTCTTGTCAATTTAAAGATATAAGATTCTTTAAATGATTAAAAAAAATATATTATAAATTCAATCACTCCTACTAATAAATTATTAATTAATATTTATTGATTAAAAAAATACTTATACTAATTTAGTCTGAATAGAATAATTAGATTCTAGAGTCGACCTGCAGGCATGCAAGCTTCTAGAGATCCGTCAACATGGTGGAGCACGACACTCTCGTCTACTCCAAGAATATCAAAGATACAGTCTCAGAAGACCAAAGGGCTATTGAGACTTTTCAACAAAGGGTAATATCGGGAAACCTCCTCGGATTCCATTGCCCAGCTATCTGTCACTTCATCAAAAGGACAGTAGAAAAGGAAGGTGGCACCTACAAATGCCATCATTGCGATAAAGGAAAGGCTATCGTTCAAGATGCCTCTGCCGACAGTGGTCCCAAAGATGGACCCCCACCCACGAGGAGCATCGTGGAAAAAGAAGACGTTCCAACCACGTCTTCAAAGCAAGTGGATTGATGTGATATCTCCACTGACGTAAGGGATGACGCACAATCCCACTATCCTTCGCAAGACCCTTCCTCTATATAAGGAAGTTCATTTCATTTGGAGAGGACGACCTGCAGGTCGACGGATCGATCCCCGGGAGCTTGGCGCTCGTCCGGGGGCAATCAGATATGAAAAAGCCTGAACTCACCGCGACGTCTGTCGAGAAGTTTCTGATCGAAAAGTTCGACAGCGTCTCCGACCTGATGCAGCTCTCGGAGGGCGAAGAATCTCGTGCTTTCAGCTTCGATGTAGGAGGGCGTGGATATGTCCTGCGGGTAAATAGCTGCGCCGATGGTTTCTACAAAGATCGTTATGTTTATCGGCACTTTGCATCGGCCGCGCTCCCGATTCCGGAAGTGCTTGACATTGGGGAATTCAGCGAGAGCCTGACCTATTGCATCTCCCGCCGTGCACAGGGTGTCACGTTGCAAGACCTGCCTGAAACCGAACTGCCCGCTGTTCTGCAGCCGGTCGCGGAGGCCATGGATGCGATCGCTGCGGCCGATCTTAGCCAGACGAGCGGGTTCGGCCCATTCGGACCGCAAGGAATCGGTCAATACACTACATGGCGTGATTTCATATGCGCGATTGCTGATCCCCATGTGTATCACTGGCAAACTGTGATGGACGACACCGTCAGTGCGTCCGTCGCGCAGGCTCTCGATGAGCTGATGCTTTGGGCCGAGGACTGCCCCGAAGTCCGGCACCTCGTGCACGCGGATTTCGGCTCCAACAATGTCCTGACGGACAATGGCCGCATAACAGCGGTCATTGACTGGAGCGAGGCGATGTTCGGGGATTCCCAATACGAGGTCGCCAACATCTTCTTCTGGAGGCCGTGGTTGGCTTGTATGGAGCAGCAGACGCGCTACTTCGAGCGGAGGCATCCGGAGCTTGCAGGATCGCCGCGGCTCCGGGCGTATATGCTCCGCATTGGTCTTGACCAACTCTATCAGAGCTTGGTTGACGGCAATTTCGATGATGCAGCTTGGGCGCAGGGTCGATGCGACGCAATCGTCCGATCCGGAGCCGGGACTGTCGGGCGTACACAAATCGCCCGCAGAAGCGCGGCCGTCTGGACCGATGGCTGTGTAGAAGTACTCGCCGATAGTGGAAACCGACGCCCCAGCACTCGTCCGAGGGCAAAGGAATAGAGTAGATGCCGACCGAACAAGAGCTGATTTCGAGAACGCCTCAGCCAGCAACTCGCGCGAGCCTAGCAAGGCAAATGCGAGAGAACGGCCTTACGCTTGGTGGCACAGTTCTCGTCCACAGTTCGCTAAGCTCGCTCGGCTGGGTCGCGGGAGGGCCGGTCGCAGTGATTCAGGCCCTTCTGGATTGTGTTGGTCCCCAGGGCACGATTGTCATGCCCACGCACTCGGGTGATCTGACTGATCCCGCAGATTGGAGATCGCCGCCCGTGCCTGCCGATTGGGTGCAGATCCGTCGACCTGCAGATCGTTCAAACATTTGGCAATAAAGTTTCTTAAGATTGAATCCTGTTGCCGGTCTTGCGATGATTATCATATAATTTCTGTTGAATTACGTTAAGCATGTAATAATTAACATGTAATGCATGACGTTATTTATGAGATGGGTTTTTATGATTAGAGTCCCGCAATTATACATTTAATACGCGATAGAAAACAAAATATAGCGCGCAAACTAGGATAAATTATCGCGCGCGGTGTCATCTATGTTACTAGATCTCTAGAAGCTTGAGTATTCTATAGTGTCACCTAAATAGCTTGGCGTAATCATGGTCATAGCTGTTTCCTGTGTGAAATTGTTATCCGCTCACAATTCCACACAACATACGAGCCGGAAGCATAAAGTGTAAAGCCTGGGGTGCCTAATGAGTGAGCTAACTCACATTAATTGCGTTGCGCTCACTGCCCGCTTTCCAGTCGGGAAACCTGTCGTGCCAGCTGCTTCCGCTTCCTCGCTCACTGACTCGCTGCGCTCGGTCGTTCGGCTGCGGCGAGCGGTATCAGCTCACTCAAAGGCGGTAATACGGTTATCCACAGAATCAGGGGATAACGCAGGAAAGAACATGTGAGCAAAAGGCCAGCAAAAGGCCAGGAACCGTAAAAAGGCCGCGTTGCTGGCGTTTTTCCATAGGCTCCGCCCCCCTGACGAGCATCACAAAAATCGACGCTCAAGTCAGAGGTGGCGAAACCCGACAGGACTATAAAGATACCAGGCGTTTCCCCCTGGAAGCTCCCTCGTGCGCTCTCCTGTTCCGACCCTGCCGCTTACCGGATACCTGTCCGCCTTTCTCCCTTCGGGAAGCGTGGCGCTTTCTCATAGCTCACGCTGTAGGTATCTCAGTTCGGTGTAGGTCGTTCGCTCCAAGCTGGGCTGTGTGCACGAACCCCCCGTTCAGCCCGACCGCTGCGCCTTATCCGGTAACTATCGTCTTGAGTCCAACCCGGTAAGACACGACTTATCGCCACTGGCAGCAGCCACTGGTAACAGGATTAGCAGAGCGAGGTATGTAGGCGGTGCTACAGAGTTCTTGAAGTGGTGGCCTAACTACGGCTACACTAGAAGAACAGTATTTGGTATCTGCGCTCTGCTGAAGCCAGTTACCTTCGGAAAAAGAGTTGGTAGCTCTTGATCCGGCAAACAAACCACCGCTGGTAGCGGTGGTTTTTTTGTTTGCAAGCAGCAGATTACGCGCAGAAAAAAAGGATCTCAAGAAGATCCTTTGATCTTTTCTACGGGGTCTGACGCTCAGTGGAACGAAAACTCACGTTAAGGGATTTTGGTCATGAGATTATCAAAAAGGATCTTCACCTAGATCCTTTTAAATTAAAAATGAAGTTTTAAATCAATCTAAAGTATATATGAGTAAACTTGGTCTGACAGTTACCAATGCTTAATCAGTGAGGCACCTATCTCAGCGATCTGTCTATTTCGTTCATCCATAGTTGCCTGACTCCCCGTCGTGTAGATAACTACGATACGGGAGGGCTTACCATCTGGCCCCAGTGCTGCAATGATACCGCGAGACCCACGCTCACCGGCTCCAGATTTATCAGCAATAAACCAGCCAGCCGGAAGGGCCGAGCGCAGAAGTGGTCCTGCAACTTTATCCGCCTCCATCCAGTCTATTAATTGTTGCCGGGAAGCTAGAGTAAGTAGTTCGCCAGTTAATAGTTTGCGCAACGTTGTTGCCATTGCTACAGGCATCGTGGTGTCACGCTCGTCGTTTGGTATGGCTTCATTCAGCTCCGGTTCCCAACGATCAAGGCGAGTTACATGATCCCCCATGTTGTGCAAAAAAGCGGTTAGCTCCTTCGGTCCTCCGATCGTTGTCAGAAGTAAGTTGGCCGCAGTGTTATCACTCATGGTTATGGCAGCACTGCATAATTCTCTTACTGTCATGCCATCCGTAAGATGCTTTTCTGTGACTGGTGAGTACTCAACCAAGTCATTCTGAGAATAGTGTATGCGGCGACCGAGTTGCTCTTGCCCGGCGTCAATACGGGATAATACCGCGCCACATAGCAGAACTTTAAAAGTGCTCATCATTGGAAAACGTTCTTCGGGGCGAAAACTCTCAAGGATCTTACCGCTGTTGAGATCCAGTTCGATGTAACCCACTCGTGCACCCAACTGATCTTCAGCATCTTTTACTTTCACCAGCGTTTCTGGGTGAGCAAAAACAGGAAGGCAAAATGCCGCAAAAAAGGGAATAAGGGCGACACGGAAATGTTGAATACTCATACTCTTCCTTTTTCAATATTATTGAAGCATTTATCAGGGTTATTGTCTCATGAGCGGATACATATTTGAATGTATTTAGAAAAATAAACAAATAGGGGTTCCGCGCACATTTCCCCGAAAAGTGCCACCTGACGTCTAAGAAACCATTATTATCATGACATTAACCTATAAAAATAGGCGTATCACGAGGCCCTTTCGTCTCGCGCGTTTCGGTGATGACGGTGAAAACCTCTGACACATGCAGCTCCCGGAGACGGTCACAGCTTGTCTGTAAGCGGATGCCGGGAGCAGACAAGCCCGTCAGGGCGCGTCAGCGGGTGTTGGCGGGTGTCGGGGCTGGCTTAACTATGCGGCATCAGAGCAGATTGTACTGAGAGTGCACCATATGCGGTGTGAAATACCGCACAGATGCGTAAGGAGAAAATACCGCATCAGGAAATTGTAAGCGTTAATATTTTGTTAAAATTCGCGTTAAATTTTTGTTAAATCAGCTCATTTTTTAACCAATAGGCCGAAATCGGCAAAATCCCTTATAAATCAAAAGAATAGACCGAGATAGGGTTGAGTGTTGTTCCAGTTTGGAACAAGAGTCCACTATTAAAGAACGTGGACTCCAACGTCAAAGGGCGAAAAACCGTCTATCAGGGCGATGGCCCACTACGTGAACCATCACCCTAATCAAGTTTTTTGGGGTCGAGGTGCCGTAAAGCACTAAATCGGAACCCTAAAGGGAGCCCCCGATTTAGAGCTTGACGGGGAAAGCCGGCGAACGTGGCGAGAAAGGAAGGGAAGAAAGCGAAAGGAGCGGGCGCTAGGGCGCTGGCAAGTGTAGCGGTCACGCTGCGCGTAACCACCACACCCGCCGCGCTTAATGCGCCGCTACAGGGCGCGTCCATTCGCCATTCAGGCTGCGCAACTGTTGGGAAGGGCGATCGGTGCGGGCCTCTTCGCTATTACGCCAGCTGGCGAAAGGGGGATGTGCTGCAAGGCGATTAAGTTGGGTAACGCCAGGGTTTTCCCAGTCACGACGTTGTAAAACGACGGCCAGTGAATTGTAATACGACTCACTATA
