# Supplementary material for: An embryo lethal transgenic line manifests global expression changes and elevated protein/oil ratios in heterozygous soybean plants
Source: PLoS One. 2020 Jun 9;15(6):e0233721. doi: 10.1371/journal.pone.0233721 (PMC7282645; doi:10.1371/journal.pone.0233721)

Loaded in order from left to right
φX174 HaeIII marker (Invitrogen, 15611-015)
Stained with 10 mg/ml ethidium bromide and imaged with AlphaImagerHP (Alpha Innotech)

Gel #1B Bottom
PCR #1B 2/8-9/16
Used for S3 Fig panel B, bottom
Generation 2

Cyc: Cycling control
NC: Non-cycling control

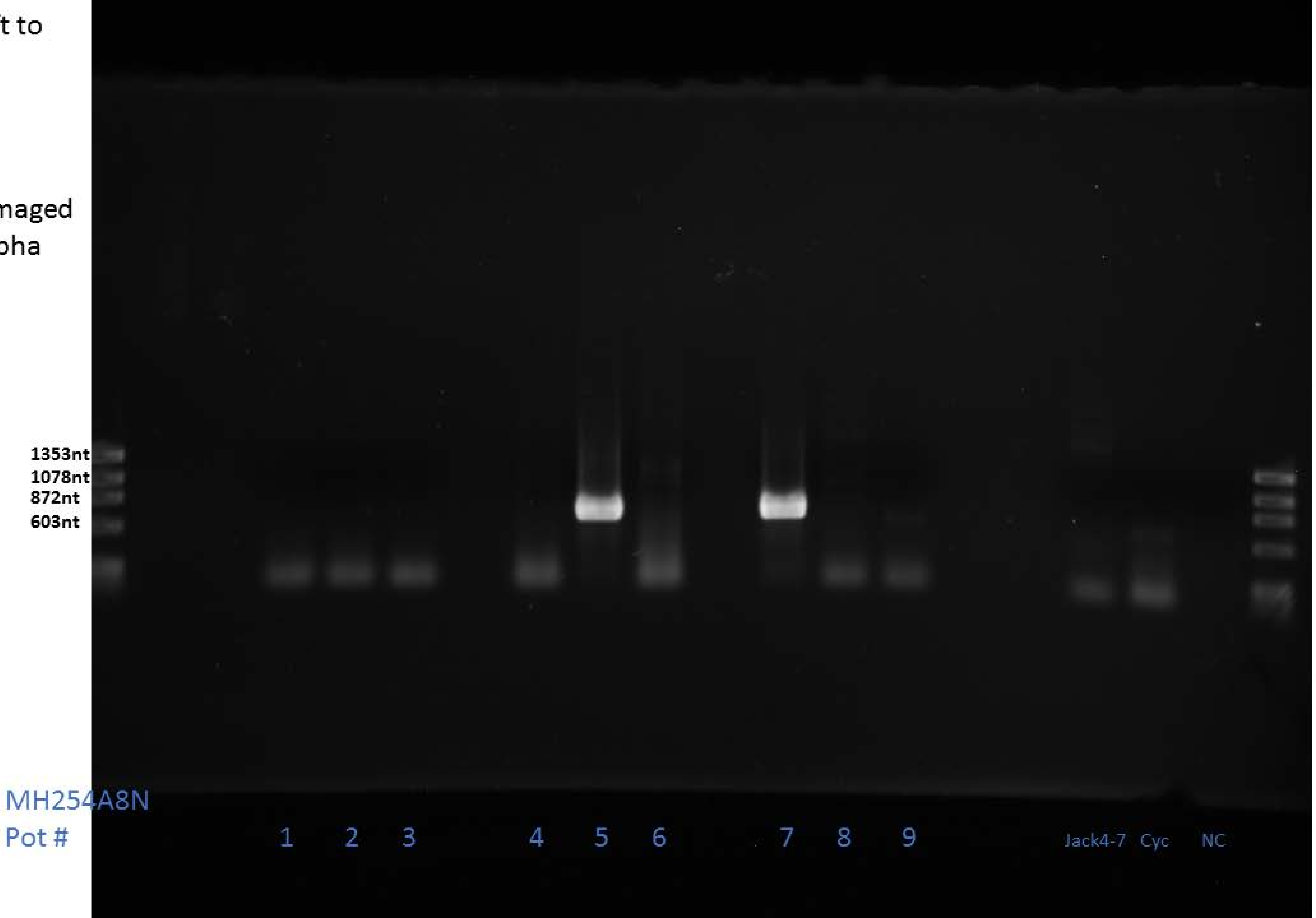

Loaded in order from left to right
ϕX174 HaeIII marker (Invitrogen, 15611-015)
Stained with 10 mg/ml ethidium bromide and imaged with AlphaImagerHP (Alpha Innotech)

Gel #1B Top
PCR #1B 2/8-9/16
Used for S3 Fig panel B, top
Generation 2

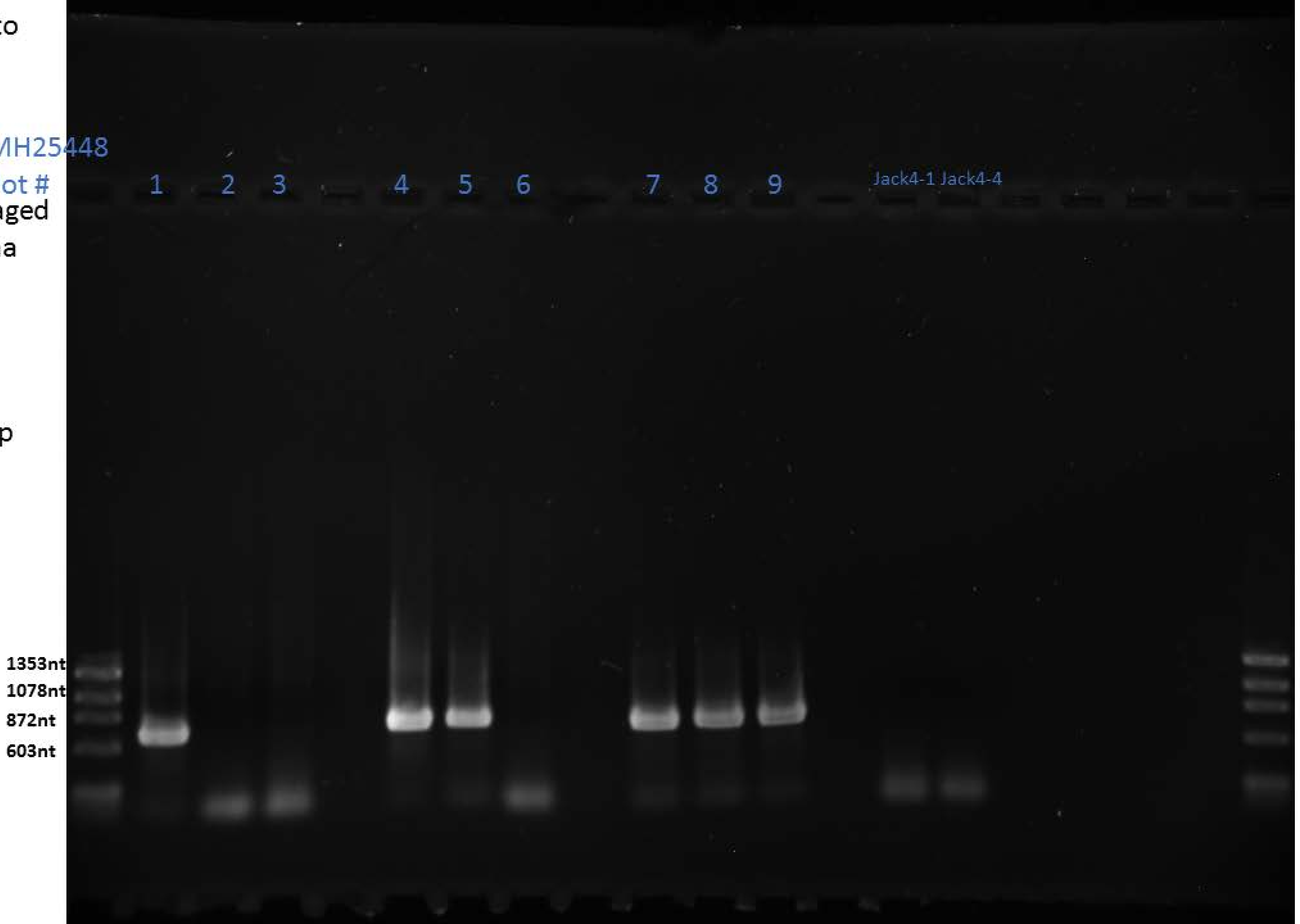

Loaded in order from left to right
ϕX174 HaeIII marker (Invitrogen, 15611-015)
Stained with 10 mg/ml ethidium bromide and imaged with AlphaImagerHP (Alpha Innotech)

Gel #1C Bottom
PCR #1C, 8/4-5/16
Used for S3 Fig panel C, middle
Generation 3

Line and pot # shown
Cyc: Cycling control
NC: Non-cycling control

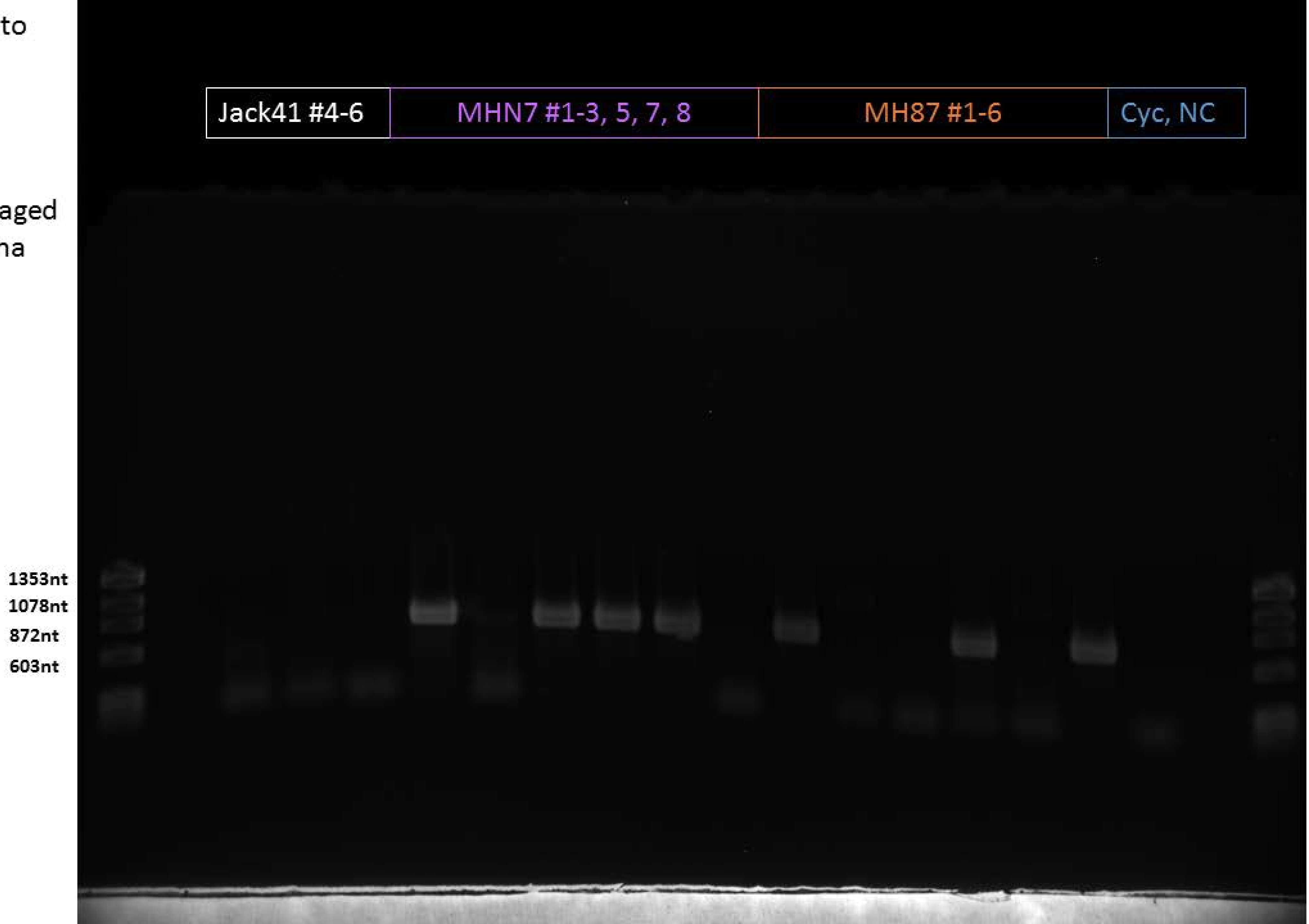

Loaded in order from left to right
ϕX174 HaeIII marker (Invitrogen, 15611-015)
Stained with 10 mg/ml ethidium bromide and imaged with AlphaImagerHP (Alpha Innotech)

Gel #1C top
PCR #1C, 8/4-5/16
Used for S3 Fig panel C, top
Generation 3

Line and pot # shown
*MH88, pot #2 did not work and was repeated later

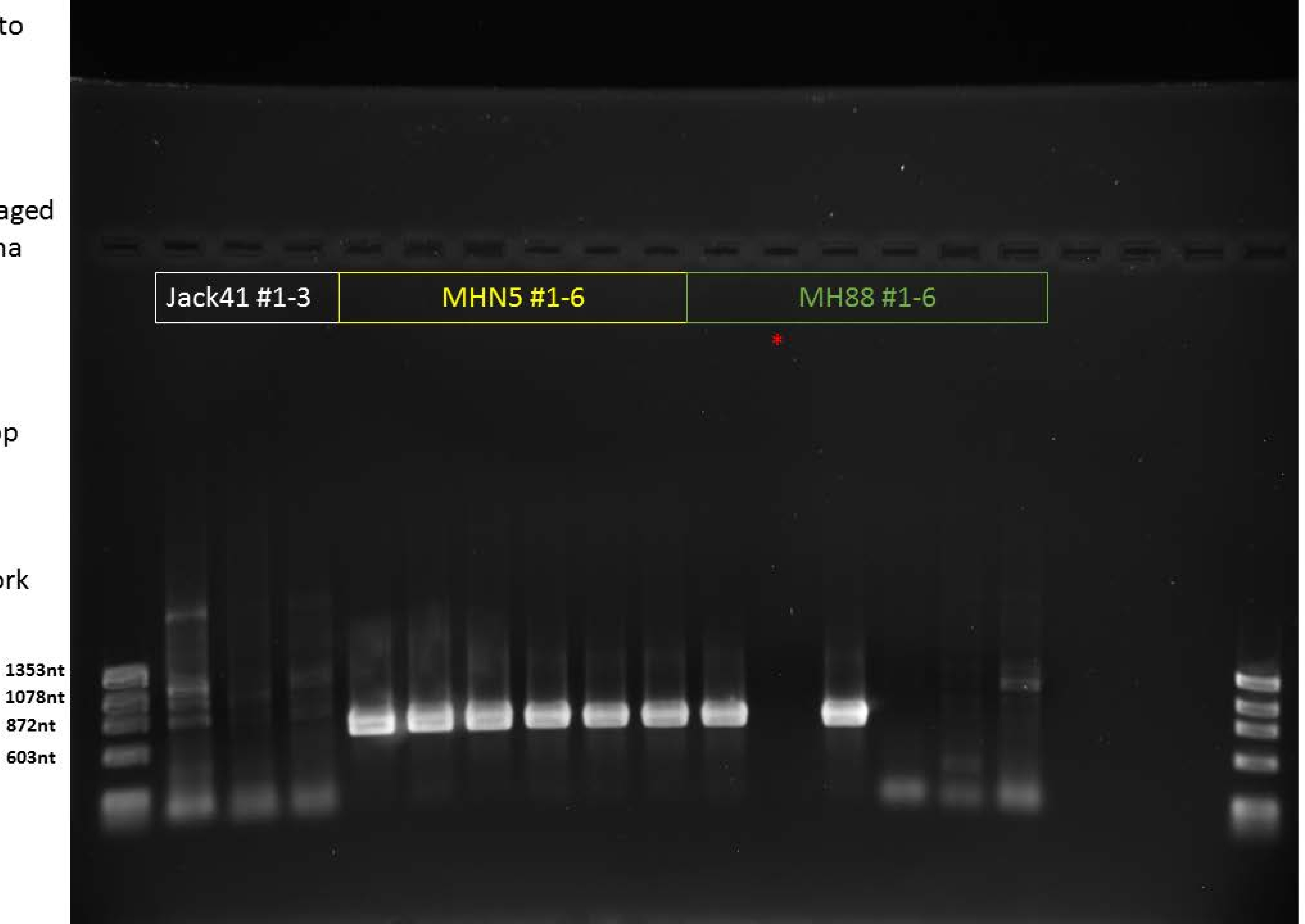

Loaded in order from left to right
ϕX174 HaeIII marker (Invitrogen, 15611-015)
Stained with 10 mg/ml ethidium bromide and imaged with AlphaImagerHP (Alpha Innotech)

Gel #2C
PCR #2C, 8/9-10/16
Used for S3 Fig panel C, bottom
Generation 3

Line and pot # shown
Cyc: Cycling control
NC: Non-cycling control

*MH88, pot #2 repeated from Gel #1C top where it did not work

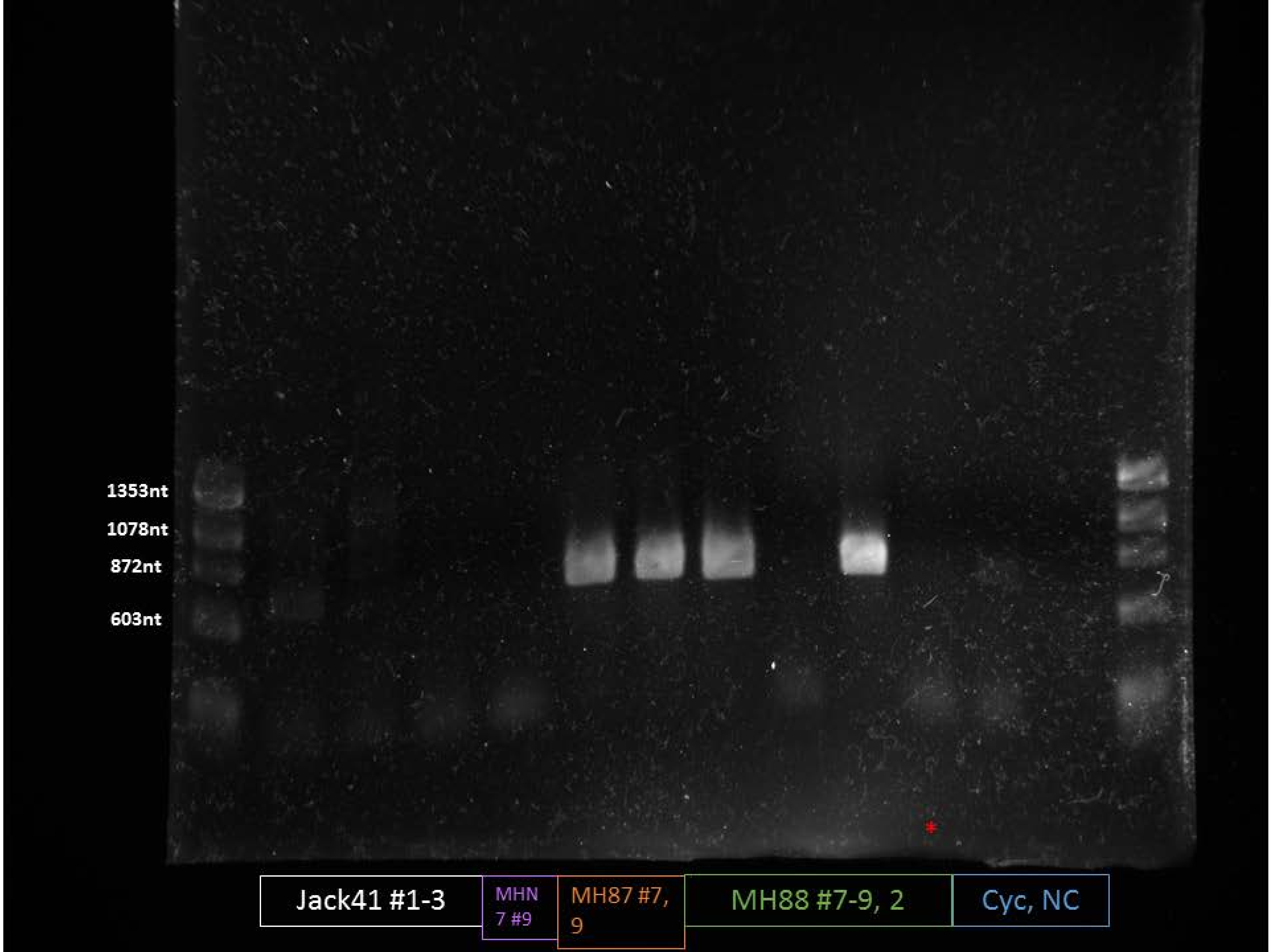

Loaded in order from left to right
ϕX174 HaeIII marker (Invitrogen, 15611-015)
Stained with 10 mg/ml ethidium bromide and imaged with AlphaImagerHP (Alpha Innotech)

Gel #12a (top)
Run 9/30/15 from PCR #9
Used for S3 Fig panel A, top
Generation 1

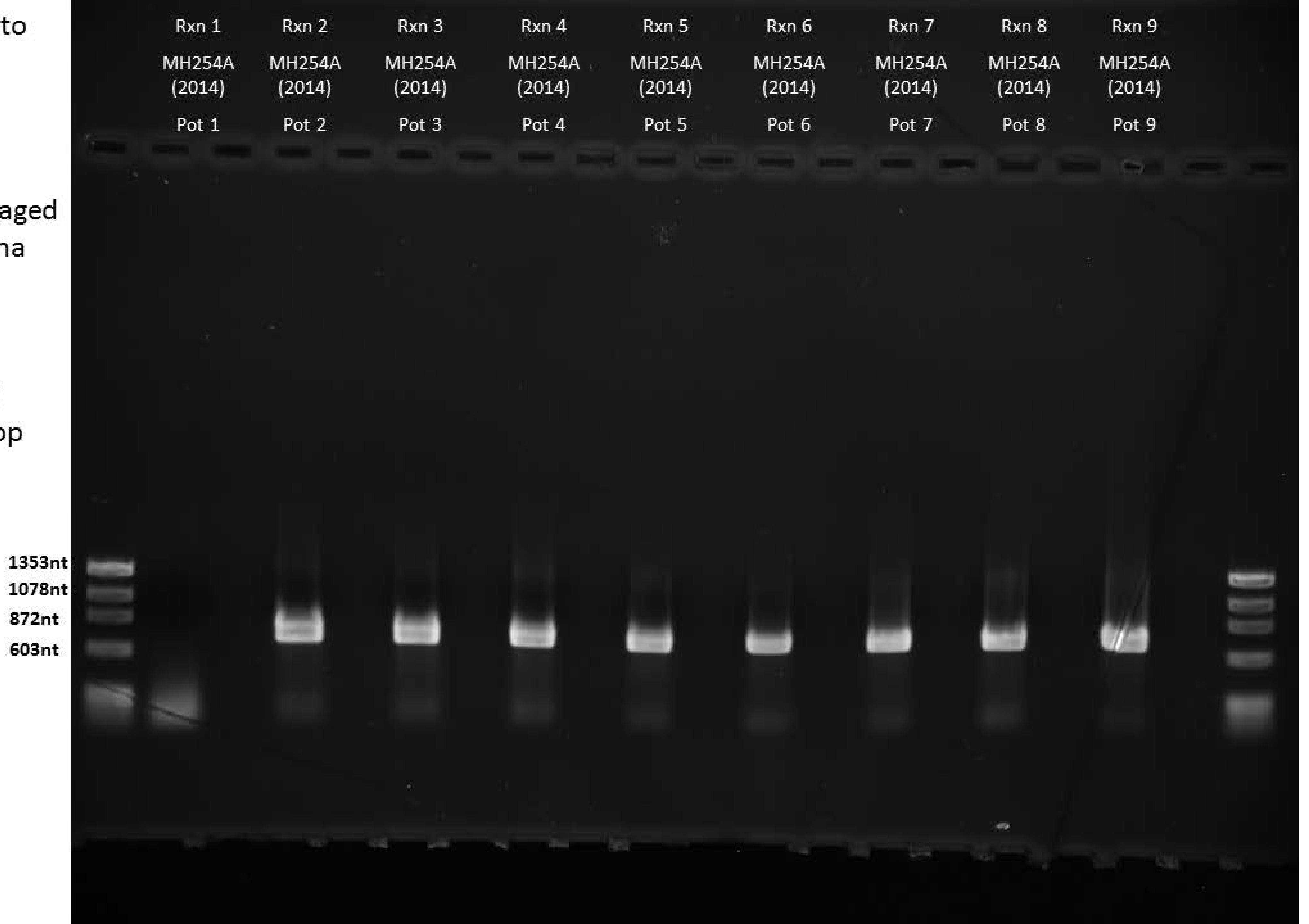

Loaded in order from left to right
ϕX174 HaeIII marker (Invitrogen, 15611-015)
Stained with 10 mg/ml ethidium bromide and imaged with AlphaImagerHP (Alpha Innotech)

Gel #12b (bottom)
Run 9/30/15 from PCR #9
Used for S3 Fig panel A, bottom
Generation 1

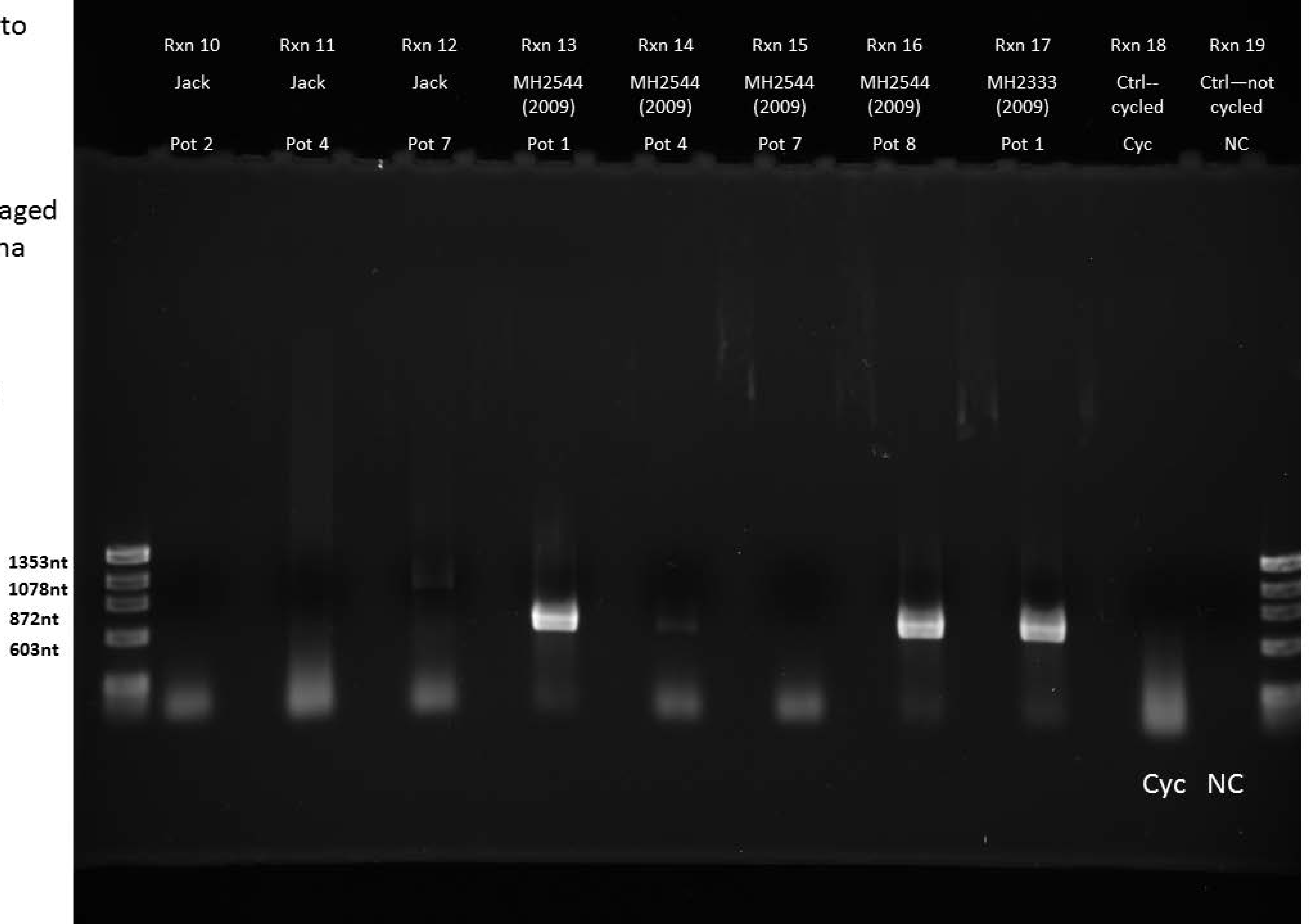

Supplement: S1 Raw images — (PDF) [file pone.0233721.s021.pdf]
